# Vitamin B_12_ is not shared by all marine prototrophic bacteria with their environment

**DOI:** 10.1038/s41396-023-01391-3

**Published:** 2023-03-13

**Authors:** Sabiha Sultana, Stefan Bruns, Heinz Wilkes, Meinhard Simon, Gerrit Wienhausen

**Affiliations:** 1 Institute for Chemistry and Biology of the Marine Environment, University of Oldenburg, Carl von Ossietzky Str. 9-11, D-26129 Oldenburg, Germany; 2 Helmholtz Institute for Functional Marine Biodiversity at the University of Oldenburg (HIFMB), Ammerländer Heerstraße 231, D-26129 Oldenburg, Germany; 3 Institute for Medical Microbiology and Virology, Carl von Ossietzky University Oldenburg, D-26129 Oldenburg, Germany

**Keywords:** Water microbiology, Microbial ecology, Symbiosis, Microbial ecology, Metabolomics

## Abstract

Vitamin B_12_ (cobalamin, herein B_12_) is an essential cofactor involved in amino acid synthesis and carbon resupply to the TCA cycle for most prokaryotes, eukaryotic microorganisms, and animals. Despite being required by most, B_12_ is produced by only a minor fraction of prokaryotes and therefore leads to complex interaction between prototrophs and auxotrophs. However, it is unknown how B_12_ is provided by prototrophs to auxotrophs. In this study, 33 B_12_ prototrophic alphaproteobacterial strains were grown in co-culture with *Thalassiosira pseudonana*, a B_12_ auxotrophic diatom, to determine the bacterial ability to support the growth of the diatom by sharing B_12_. Among these strains, 18 were identified to share B_12_ with the diatom, while nine were identified to retain B_12_ and not support growth of the diatom. The other bacteria either shared B_12_ with the diatom only with the addition of substrate or inhibited the growth of the diatom. Extracellular B_12_ measurements of B_12_-provider and B_12_-retainer strains confirmed that the cofactor could only be detected in the environment of the tested B_12_-provider strains. Intracellular B_12_ was measured by LC-MS and showed that the concentrations of the different B_12_-provider as well as B_12_-retainer strains differed substantially. Although B_12_ is essential for the vast majority of microorganisms, mechanisms that export this essential cofactor are still unknown. Our results suggest that a large proportion of bacteria that can synthesise B_12_ *de novo* cannot share the cofactor with their environment.

## Introduction

Vitamin B_12_ (cobalamin, herein B_12_) is a water-soluble cobalt-containing compound and is required by the vast majority of prokaryotic and about half of the eukaryotic marine microorganisms that are isolated or genome sequenced [1]. B_12_ functions as coenzyme for the methylcobalamin-dependent methionine synthase and adenosylcobalamin-dependent methylmalonyl-CoA mutase, which are involved in amino acid synthesis and carbon resupply to the TCA cycle, respectively [2, 3]. However, *de novo* synthesis can only be carried out by a minor fraction of prokaryotes [1, 4, 5]. More than 30 genes are required for the complete biosynthesis of this important cofactor, which makes up about 1% of an average bacterial genome [4, 6]. This is an energetically and metabolically expensive biosynthetic process, which may explain why considerably fewer than half of all prokaryotes encode the genes for complete biosynthesis of B_12_ or other cobamides [1, 4, 7, 8]. To gain an evolutionary benefit in an environment where there is still a sustainable supply from distinct substrates or growth factors such as vitamins, loss of biosynthetic genes often occurs in bacteria, leading to a reduction in the size of the genome, known as genome streamlining [9, 10]. This process is believed to reduce the metabolic cost and thus provide a selective advantage, as long as sufficient quantities of the essential compound are freely available. Less than 10% of soil bacteria are capable of *de novo* synthesise of B_12_ [8]. In marine ecosystems only select heterotrophic bacteria and Thaumarchaeota can produce it [11] and the share of B_12_ prototrophs can be as low as one-fifth of the bacterial community, so that the vast majority of microorganisms depend on the cofactor [7]. This gap between supply and demand of B_12_ can result in complex microbial interactions between prokaryotes and eukaryotes [12–16]. Approximately half of known phytoplankton encode the B_12_-dependent methionine synthase (*metH*) [17], which is why they require this pivotal cofactor from the environment. Concentrations of dissolved B_12_ undergo strong fluctuations in the sea, are in some cases below the detection limit of a few pM, and their presence has been shown to influence marine microbial communities [11, 18–23]. In marine environments, bacteria often live in close association with phytoplankton [24, 25]. In several studies, the provision of B_12_ by individual heterotrophic bacteria to B_12_ auxotrophic phytoplankton in exchange for organic carbon was demonstrated [13, 14, 16, 26–29]. Yet, it is still debatable whether this exchange of metabolic products represents a mutualistic symbiosis between B_12_ prototrophs and B_12_ auxotrophic, microbial eukaryotes, or whether its release is unintentional [13, 30, 31]. In fact, mechanisms that lead to the provision of the cofactor and factors that favor this exchange are still largely unknown. Despite varying lower and upper ligands attached to the corrinoid ring, B_12_-family metabolites, including cobalamin, are always larger than 1,350 Daltons. Therefore, diffusion through the cell membrane appears to be almost impossible [32]. The relatively well studied uptake of B_12_ by gram-negative bacteria, requires the binding of B_12_ to the outer membrane protein BtuB. Then, B_12_ is transported into the cell by the inner membrane protein complex TonB via an electrochemical gradient of protons [33]. The mechanism of B_12_ export, essential for microbial interactions, still remains unexplored.

Given the available knowledge, we hypothesise that not all B_12_ prototrophic bacteria share the essential cofactor with other microorganisms. Our aim is to provide first indications of the requirement of an active export mechanism in order to draw conclusions on B_12_ driven mutualistic interactions and the global provision of B_12_ in marine ecosystems.

In order to achieve this goal, we co-cultured *Thalassiosira pseudonana*, a B_12_ auxotrophic diatom, with 33 B_12_ prototrophic bacteria of the alphaproteobacterial class to test the bacterial ability to share B_12_ with other microorganisms. Furthermore, we determined intra- and extracellular B_12_ concentrations of B_12_-provider and B_12_-retainer strains by means of liquid chromatography coupled with mass spectrometry (LC-MS) [34] and studied patterns that both B_12_-provider and B_12_-retainer strains had in common.

## Methods and materials

### Identification of B_12_ prototrophs

To study the ability of heterotrophic bacteria to share B_12_ with surrounding microorganisms, we selected 33 prototrophic marine representatives. As a prerequisite, all selected bacteria had to grow on synchronized artificial seawater (syn-ASW) media (Supplementary table S1) as it promotes the growth of most phototrophic eukaryotes as well as prokaryotes with equal growth facilitating conditions. Further, the genome sequence had to be complete and accessible at IMG (integrated microbial genomes; https://img.jgi.doe.gov/cgi-bin/mer/main.cgi). The ability for *de novo* B_12_ synthesis was verified based on a complete B_12_ pathway and growth (determined by OD) in minimal medium without the addition of B_12_. The genetic verification of the B_12_ biosynthesis pathway was assumed if at least 95% of the B_12_ biosynthesis pathway of a strain was annotated (Supplementary table S2). As B_12_ auxotrophic (recipient) organism, we selected the genome sequenced diatom, *T. pseudonana* (CCMP 1335).

### B_12_ cross-feeding co-culture experiment

To establish a B_12_ deficient diatom culture, *T. pseudonana* was first cultured in F/2 media and subsequently transferred (twice) to B_12_-free syn-ASW media (supplemented with thiamine (vitamin B_1_), riboflavin (vitamin B_2_), nicotinic acid (vitamin B_3_), pantothenic acid (vitamin B_5_), pyridoxine hydrochloride (vitamin B_6_), and biotin (vitamin B_7_); 500 pM each). Once B_12_ was depleted, the final diatom pre-culture for the inoculation of the main experiment was supplemented with 10 pM B_12_ to ensure growth, yet growth was limited upon B_12_ depletion. Bacterial pre-cultures were grown in Marine Broth (MB) media at 20 °C, 100 rpm. Cultures of the late exponential growth phase were washed three times (5974 g, five minutes) with B_12_-free syn-ASW media prior to inoculation. All diatom pre-cultures and the experimental co-cultures were illuminated at 70 µE m^-2^ s^−1^ and incubated at 20 °C with a 12:12 h light-dark cycle (RUMED). The diatom-bacteria co-cultures were grown at three varying conditions. First, bacterial isolates were co-cultured with the diatom *T. pseudonana* without further additions to determine whether growth of *T. pseudonana* upon bacterial B_12_ release is enabled. Second, to eliminate the possibility that individual bacterial isolates are unable to utilize diatom derived organic carbon and thus are unable to share B_12_, the co-culture was supplemented with an organic carbon mixture (120 µM C), containing glucose, glutamate, and acetate (each substrate at 40 µM C). Third, B_12_ (1 nM) was added to the bacteria-diatom co-culture, to ensure that the growth of the diatom was not inhibited by the bacteria, thus resulting in a false consideration as B_12_-retainer. Alongside each experimental run, a negative control, axenic *T. pseudonana* grown without B_12_ addition and a positive control, *T. pseudonana* grown with the addition of 1 nM B_12_ was considered. All treatments were run as triplicates. To ensure that only the bacterially provided B_12_, but not the possible provision of methionine, enables the growth of *T. pseudonana* in co-culture, we cultivated *T. pseudonana* with only the addition of methionine and did not observe growth (Supplementary Fig. S1). For all co-culture treatments containing bacteria, the initial bacterial inoculum was calculated to be at 500,000 cells ml^−1^ (based on flow cytometric cell counts), initial *T. pseudonana* cells were estimated to be about 4,000 cells ml^−1^ (microscopic enumeration). Bacterial-diatom co-cultures were illuminated at 70 µE m^−2^ s^−1^ and incubated at 20 °C with a 12:12 h light-dark cycle. Growth of *T. pseudonana* was determined throughout the experiment by means of relative fluorescence (TD 700 fluorometer, Turner Designs, California, USA). Samples for diatom and bacterial cell count were collected after inoculation and during the early stationary growth phase of *T. pseudonana*. For bacterial cell counts, samples were fixed with GDA at a final concentration of 1%, incubated at 4 °C for 30 min, and stored at −20 °C until enumeration by flow cytometry [35]. Diatom samples for cell enumeration were fixed with lugol (final concentrations of 0.15% iodine and 0.29% potassium iodide) and stored at 4 °C until further analysis.

### Enumeration of bacteria and diatom

Prior to counting with the flow cytometer, bacterial cells were detached from the diatom cells using glass beads (2.3 mm) and ultrasonication (35 °C, 70%, 4 ×5 minutes, Sonorex digital 10P, Bandelin, Berlin, Germany) following by a short vortexing step (2 × 2 seconds, Vortex Genie2, Scientific Industries Inc., New York, USA) after each ultrasonic interval. This method was a further development of the detachment method described elsewhere [36]. Afterwards, bacterial cells were stained with SybrGreen I and enumerated by flow cytometry (BD Accuri C6, BD biosciences, Franklin Lakes NJ, USA) as described elsewhere [35]. Diatom samples, fixed with lugol, were loaded on a hemocytometer and enumerated by microscopy (AXIO, Lab.A1, objective lens Carl Zeiss, 40x).

### Measurement of intra- and extracellular B_12_ concentrations

Intracellular B_12_ concentrations of 20 bacterial strains were measured using LC-MS. Selected strains were grown in B_12_-free syn-ASW media (see above) and supplemented with an organic substrate mix (30 mM C) containing glucose, acetate, and glutamate (each having 10 mM C). Cell pellets of 2 × 50 ml culture were harvested from each replicate during the late exponential or early stationary growth phase, monitored by means of optical density (OD; Tables 1 and 2, Supplementary Fig. S2 and S3). The samples were then washed twice with B_12_-free syn-ASW medium (3,213 g, five minutes at 4 °C) and cell pellets were stored at −20 °C until further analysis. Alongside, samples for bacterial cell counts were withdrawn, fixed with GDA (final concentration 1%), and enumerated by flow cytometry (see above).

**Table 1:** Intra- and extracellular vitamin B_12_ concentrations obtained from B_12_-provider strains when grown in mono-culture and growth rates of *T. pseudonana* in co-culture with respective bacterial strain without and with the addition of B_12_ and with the addition of B_12_ in monoculture.

B_12_ provider strains	Strain designation	Cell collection method used	Intracellular B_12_ molecules/ cell	Extracellular B_12_ molecules / cell	Growth rate of *T. pseudonana* grown in co-culture without addition of B_12_ (day)	Growth rate of *T. pseudonana* grown in co-culture with addition of B_12_ (day)	Growth rate of *T. pseudonana* only with addition of B_12_ (day)
*Antarctobacter heliothermus* EL-219	DSM 11445	Cell pellet	753 ± 232	N/A	3.7 ± 1.4	2.6 ± 0.04	2.8 ± 0.02
*Dinoroseobacter shibae*	DSM 16493	Filtration	7,194 ± 1,105	N/A	9.6 ± 0.9	8.8 ± 0.3	8.1 ± 0.1
*Marinovum algicola* FF3	DSM 10251	Cell pellet	7,171 ± 792	936 ± 363	2.8 ± 0.04	2.8 ± 0.04	2.8 ± 0.02
*Nautella italica* R11	DSM 26436	Cell pellet	1,986 ± 273	N/A	5.6 ± 0.2	2.7 ± 0.02	2.8 ± 0.02
*Phaeobacter inhibens*	DSM 17395	Cell pellet	2,821 ± 540	11 ± 3	10.3 ± 1.1	8.2 ± 1.1	8.1 ± 0.1
*Phaeobacter inhibens* T5	DSM 16374	Cell pellet	664 ± 88	N/A	5.6 ± 0.5	2.5 ± 0.05	3.4 ± 0.4
*Ponticoccus litoralis* CL-GR66	DSM 18986	Cell pellet	4,622 ± 2,227	N/A	6.5 ± 1.2	3.8 ± 0.04	3.4 ± 0.4
*Aliiroseovarius crassostreae* CV919-312	DSM 16950	Cell pellet	26,619 ± 13,140	N/A	5.8 ± 1.7	4.7 ± 0.9	3.4 ± 0.4
*Roseovarius mucosus* DFL-24	DSM 17069	Cell pellet	4,028 ± 708	N/A	6.0 ± 0.1	3.8 ± 0.06	3.4 ± 0.4
*Silicibacter* sp.	TM1040	Cell pellet	15,214 ± 3,566	N/A	7.7 ± 0.5	4.3 ± 1.0	3.4 ± 0.4
*Sulfitobacter* sp.	DFL-14	Cell pellet	1546 ± 124	N/A	5.9 ± 0.8	5.4 ± 1.2	3.4 ± 0.4
*Sulfitobacter* sp.	M22	Filtration	13,982 ± 6,646	N/A	2.9 ± 0.2	2.7 ± 0.1	2.8 ± 0.02

*N/A not available.

**Table 2:** Intra- and extracellular vitamin B_12_ concentrations obtained from B_12_-retainer strains when grown in mono-culture and growth rates of *T. pseudonana* in co-culture with respective bacterial strain with the addition of B_12_ and with the addition of B_12_ in mono-culture.

B_12_ retainer strains	Strain designation	Cell collection method used	Intracellular B_12_ molecules/ cell	Extracellular B_12_ molecules / cell	Growth rate of *T. pseudonana* grown in co-culture without addition of B_12_ (day)	Growth rate of *T. pseudonana* grown in co-culture with addition of B_12_ (day)	Growth rate of *T. pseudonana* only with addition of B_12_ (day)
*Pseudodongicola xiamenensis* Y2	DSM 18339	Filtration	2,656 ± 421	ND	N/A	7.2 ± 1.5	8.1 ± 0.1
*Jannaschia helgolandensis* Hel10	DSM 14858	Filtration	ND	ND	N/A	2.3 ± 0.03	3.8 ± 0.9
*Loktanella salsilacus* R-8904	DSM 16199	Cell pellet	671 ± 281	N/A	N/A	2.8 ± 0.02	2.8 ± 0.02
*Phaeobacter gallaeciensis* BS 107	DSM 26640	Filtration	ND	N/A	N/A	2.8 ± 0.01	2.8 ± 0.02
*Sulfitobacter* sp.	DFL-23	Filtration	ND	N/A	N/A	7.5 ± 0.1	8.1 ± 0.1
*Sulfitobacter* sp.	M39	Filtration	4,599 ± 122	N/A	N/A	2.7 ± 0.04	2.8 ± 0.02
*Loktanella* sp.	M215	Cell pellet	ND	N/A	N/A	2.4 ± 0.00	3.8 ± 0.9
*Sulfitobacter* sp.	M220	Cell pellet	4,558 ± 106	N/A	N/A	2.8 ± 0.08	2.8 ± 0.02

*ND not detected, *N/A* not available.

To also analytically analyse whether B_12_ prototrophic bacteria share B_12_, we sampled the exometabolome of four representative bacterial strains. We chose two isolates (*Marinovum algicola* FF3 DSM 10251, *Phaeobacter inhibens* DSM 17395) that promoted the growth of *T. pseudonana* in co-culture, while the other two isolates (*Pseudodongicola xiamenensis* DSM 18339, *Jannaschia helgolandensis* DSM 14858) did not. The isolates were grown as described above and growth was monitored by OD. The exudate was collected by filtering the culture with 0.2 µm filter (Sartorius, Minisart syringe filter) during late exponential or early stationary growth phase. Samples were stored at -20 °C until further analysis.

Bacterial cell pellets for intracellular B_12_ analysis were extracted with bead beating, as described elsewhere [37]. To assess the recoveries, the work-up procedure was performed with known amounts of reference standards of the respective vitamins, and the amounts after the work-up were compared to the theoretical amounts without losses for each analyte. Recoveries of the different B_12_ forms (cyano-, adenosyl-, methyl- and hydroxycobalamin) were 97-99%. Extracellular B_12_ was concentrated on a solid phase extraction column (HLB, 1 g, Macherey-Nagel) at pH 6 and eluted with methanol [38]. The solvent extracts were dried down under nitrogen stream and redissolved in 200 µl of water. Concentrations of individual intra- and extracellular B_12_ forms were analyzed by LC-MS as described elsewhere [34] and summarized as total B_12_. For HPLC separation with an Ultimate 3000 (ThermoFisher Scientific) on a Kinetex Evo C18 column (100 × 2.1 mm, 2.6 µm pore size, Phenomenex, Torrance, CA, USA) 10 mM ammonium formate (pH 6.0) (A) and acetonitrile (B) were used with the following solvent gradient: 0–13 min 100–75% A; 13–15 min 75–0% A; 15–19 min 0% A; 19–21 min 0–100% A; 21–26 min 100% A. Parameters for selected reaction monitoring mode on a TSQ Quantum Ultra triple quadrupole mass spectrometer (ThermoFisher Scientific) can be found in supplementary table S3.

### Determination of the B_12_ requirement of *T. pseudonana*

In order to get a better insight into the B_12_ requirement of *T. pseudonana*, we grew the axenic diatom in cultures of triplicates in syn-ASW-medium with the addition of different B_12_ concentrations (5, 10, 25, 50, and 100 pM) and without any addition. All cultures were illuminated at 70 µE m^−2^ s^−1^ and incubated at 20 °C with a 12:12 h light-dark cycle. The growth was determined every two to three days by means of relative fluorescence and the determination of the cell numbers (see above).

## Results

### Growth of *T. pseudonana* at varying B_12_ concentrations

**Fig. 1:**
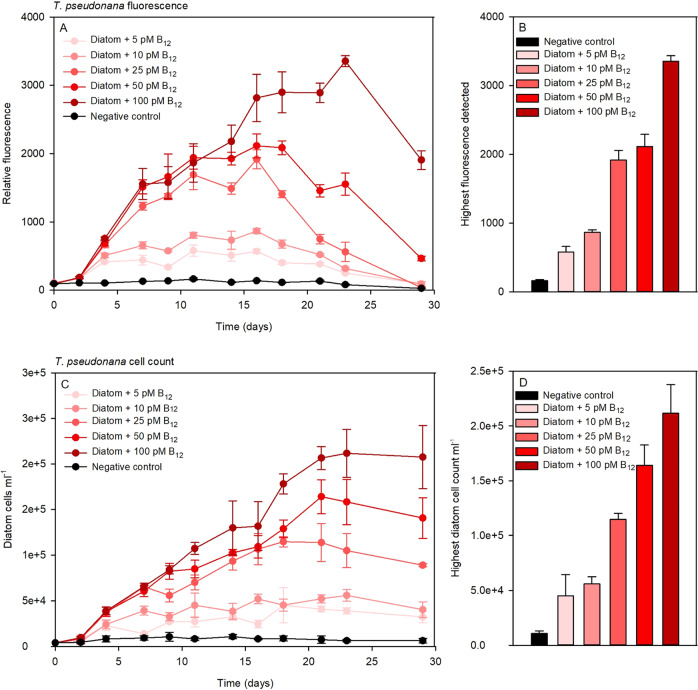
*T. pseudonana* vitamin B_12_ bioassay. Shown is the growth of *T. pseudonana* at varying B_12_ concentrations, monitored by relative fluorescence (**A**, **B**) and cell count (**C**, **D**). In **A** the Y-axis represents the relative fluorescence and in **C** the Y-axis represents diatom cell count/ml. Depicted are maximum growth determined by relative fluorescence (**B**) and cell count (**D**) of the diatom at respective B_12_ concentrations.

We observed similar growth yield patterns by adding different B_12_ concentrations both by relative fluorescence (Fig. 1A) and by cell number determination (Fig. 1C). However, especially in the later growth phase (days 18 - 29), a strong decrease in relative fluorescence occurred, whereas at the same time points microscopically counted cell numbers still increased strongly (Fig. 1A, C). Highest relative fluorescence and also *T. pseudonana* cell density was achieved with the addition of 100 pM B_12_ (Fig. 1B, D). All other measured values rank according to the added concentration. Even the addition of fairly low B_12_ concentrations (five pM) resulted in significant growth compared to the negative control, which was detected by means of relative fluorescence as well as cell enumeration.

### Growth of *T. pseudonana* in co-culture

**Fig. 2:**
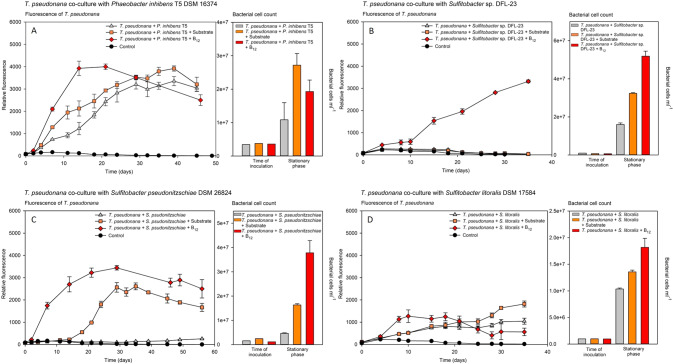
Growth of *T. pseudonana* in co-culture with B_12_ prototrophic bacteria. Representative co-cultures of *T. pseudonana* with B_12_ prototrophic bacteria that provide B_12_ (**A**), retain B_12_ (**B**), provide only when substrate is available (**C**) and likely provide B_12_ while inhibiting growth (**D**). (left panels, growth curves) Growth of *T. pseudonana* monitored by relative fluorescence unit (RFU) over time with additions of substrate mix (orange square), B_12_ (red diamond), or without addition of either (grey triangle). (Right panels; bar plots) Bacterial cell counts in co-cultures at the time of inoculation and early stationary growth phase of *T. pseudonana*. **A** *P. inhibens* T5 supports the growth of *T. pseudonana* by providing B_12_ (Further examples in Fig. S4); **B** *Sulfitobacter* sp. DFL-23 retains B_12_ and does not support growth of the diatom (Further examples in Fig. S5); **C** *S. pseudonitzschiae* provides B_12_ only with additions of a substrate mix (Further examples in Fig. S6); and **D** *S. litoralis* provides B_12_ and inhibits growth of *T. pseudonana*.

**Fig. 3:**
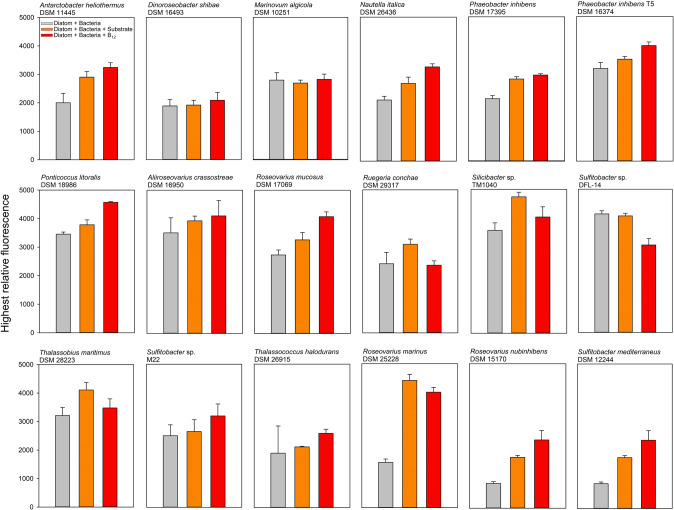
Maximum growth of *T. pseudonana* in co-culture with B_12_-provider. Bars represent the maximum relative fluorescence of *T. pseudonana* during growth in co-culture with 18 different B_12_-providers under different growth conditions (corresponding growth curves can be seen in the appendix). Grey bars represent maximum relative fluorescence of *T. pseudonana* in co-cultures without further additions, orange co-cultures with an additional substrate mix and red the co-cultures with B_12_ additions.

**Fig. 4:**
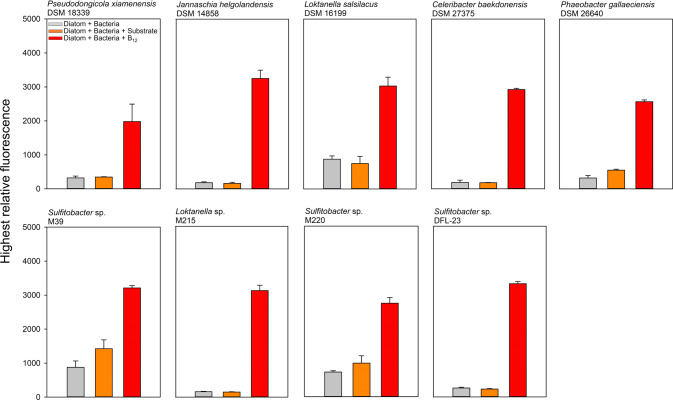
Maximum growth of *T. pseudonana* in co-culture with B_12_-retainer. Bars represent the maximum relative fluorescence of *T. pseudonana* during growth in co-culture with nine different B_12_-retainers under different growth conditions (corresponding growth curves can be seen in the appendix). Grey bars represent maximum relative fluorescence of *T. pseudonana* in co-cultures without further additions, orange co-cultures with an additional substrate mix and red the co-cultures with B_12_ additions.

To analyze the B_12_ sharing between B_12_ prototrophic bacteria and the auxotrophic diatom *T. pseudonana*, both microorganisms were co-cultured. Among 33 B_12_ prototrophic bacterial strains, 18 promoted the growth of the diatom. Growth of *T. pseudonana* in co-culture with B_12_-providing bacteria mostly achieved the same growth yield as the positive control, where the alga was grown with addition of 1 nM B_12_, however with a slightly delayed growth (Fig. 2A, Fig. 3 and Supplementary Fig. S4). In the following, this group of bacteria is referred to as B_12_-provider (Table 1). Co-cultivation with nine other B_12_ prototrophic bacteria did not result in distinct growth of the diatom, although the bacterial cell counts increased significantly over the course of the co-culture. The addition of substrate to exclude the possibility that the respective bacteria cannot utilise the diatom derived dissolved organic carbon did not lead to growth of the diatom either (Fig. 2B, Fig. 4, and Supplementary Fig. S5). However, the additional supply of B_12_ to the co-culture led to growth of *T. pseudonana*, which eliminates possible growth inhibition of the diatom induced by the tested bacteria. The group of these B_12_ prototrophic bacteria is hereafter referred to as B_12_-retainer (Table 2). Apart from the B_12_-provider and B_12_-retainer strains, co-cultivation with six additional B_12_ prototrophic bacterial isolates did not show distinct results, that would clearly favour one of the two groups. Five bacterial isolates were particularly growth-promoting when additional substrate was added to the co-culture. This observation suggests that these bacterial isolates cannot utilise the diatom derived DOM. In fact, most of these isolates (four out of five) were isolated from a source other than diatoms (Supplementary table S4). It is quite possible that these bacteria also share the synthesised B_12_ with their environment (Fig. 2C and Supplementary Fig. S6 and S7). Nevertheless, we have not considered this group for follow-up analysis. In co-cultivation with one bacterial strain, *S. litoralis*, the growth of *T. pseudonana* was inhibited under all three culture conditions. Growth yield of *T. pseudonana* remained at only half the level seen when *T. pseudonana* was grown in monoculture with the addition of B_12_ (Fig. 2D). Again, it can be assumed that *S. litoralis* shares B_12_ with the diatom, but again, we did not take this strain into account for further investigations.

### Growth characteristics of bacteria and *T. pseudonana* in co-culture

In most B_12_-provider-diatom co-cultures without supplementations of substrate or B_12_, *T. pseudonana* achieved the same growth yield as with the additional supply of B_12_, however, mostly with a slight delay in growth (Fig. 2A and Supplementary Fig. S4). Growth rates in most co-cultures were fastest with the addition of B_12_, followed by co-cultures with substrate additions and mostly slowest in co-cultures without any addition. The only exception was *Sulfitobacter* sp. DFL-14, in which the growth rate of *T. pseudonana* was fastest in co-culture without the addition of B_12_ compared to the one with B_12_ (Supplementary Fig. S4). In all cultures with B_12_-provider strains, bacterial cell counts in the late exponential or early stationary growth phase were distinctly above those of the time point of inoculation (Fig. 2A and Supplementary Fig. S4). Bacterial cell counts of all these co-cultures for the treatments with substrate and B_12_ addition were mostly in the same order of magnitude, whereas co-cultures without further additions were mostly slightly below these values. The only exception was observed for the co-cultures with *A. crassostreae* DSM 16950, in which the highest bacterial cell counts were detected in the co-culture without further additions (Supplementary Fig. S4).

In the B_12_-retainer-diatom co-cultures, we observed considerable differences in growth rates and yield in co-cultures with B_12_ addition. The significantly increased growth yield of *T. pseudonana* when co-cultured with *C. baekdonensis* DSM 27375 and B_12_ additive was very noticeable (Supplementary Fig. S5). The relative fluorescence of this co-culture was almost twice as high as compared to others. In most of the B_12_-retainer-diatom co-cultures, the detected relative fluorescence of *T. pseudonana* without any and with substrate addition was comparable to negative control values of *T. pseudonana*, when cultivated axenically without B_12_ addition. Only a slight growth of *T. pseudonana* in co-culture with the B_12_-retainers *L. salsilacus*, *Sulfitobacter* sp. M39, and *Sulfitobacter* sp. M220 was observed (Supplementary Fig. S5). Due to the low growth, which only became apparent in the later course of the growth curve, we nevertheless classified these strains as B_12_-retainers. For all B_12_-retainer-diatom co-cultures, the bacterial cell counts sampled at the stationary phase (B_12_ addition) or at the end of the experiment (no addition and substrate addition) were significantly higher than the measurements at the time of inoculation (Fig. 2B and Supplementary Fig. S5). Only in B_12_-retainer-diatom co-cultures (without any addition), *J. helgolandensis* DSM 14858, *Loktanella* sp. M215, and *P. gallaciensis*, a slight to no increases in cell numbers was detected, yet an increase in cell numbers with substrate addition was detected in all co-cultures (Supplementary Fig. S5). The bacterial strains studied, divided into the groups of B_12_-providers and B_12_-retainer, are listed in supplementary table S4 with their known habitats or isolation sites. Here it can be seen that especially bacteria of the B_12_-provider group were isolated from or are mostly living in association with eukaryotic microorganisms.

### Intra- and extracellular B_12_ concentration

Twenty of the bacteria that we identified as either B_12_-provider or B_12_-retainer were grown again in monoculture with the addition of substrate, to determine the intracellular concentration of B_12_. The growth yield of some B_12_-retainer strains was significantly lower, which is why their biomass sampling yield was significantly lower as well. Detected B_12_ concentrations were normalised against cell numbers to better distinguish between cultures with different growth rates and yields. In some cases, the intracellular B_12_ values differed immensely, with 40-fold deviations within the B_12_-provider strains. When comparing intracellular B_12_ values of individual B_12_-provider strains to their ability to impact the growth rate of *T. pseudonana* in co-culture through their release of B_12_, we cannot discern a direct correlation (Table 1). In B_12_-retainer strains, we were unable to detect B_12_ in four out of eight bacterial cultures (Table 2). Detected B_12_ values varied between 671 to 4,599 B_12_ molecules per cell. The four detected intracellular B_12_ values of B_12_-retainer strains were comparable to the average values measured for the B_12_-providers (Tables 1 and 2).

Extracellular B_12_ was measured additionally in two selected bacterial strains from the groups of B_12_-provider and B_12_-retainer, each of which exhibited a comparably high growth yield. B_12_ was detected in both B_12_-provider cultures (*M. algicola* and *P. inhibens*), while no B_12_ was measured in both B_12_-retainer cultures (*P. xiamenensis* and *J. helgolandensis*, Tables 1 and 2). Extracellular B_12_ concentrations of the two B_12_-provider strains were approximately 8 and 256 times lower than the corresponding intracellularly detected values (Table 1). However, when evaluating these values and drawing conclusions for the observations from the co-cultures, it must be considered that the values were obtained from monocultures with a significantly shortened growth phase. B_12_ production by prototrophic bacteria can vary in co-culture with the diatom, as it is known that algal metabolism upregulates bacterial production of B_12_ [13].

## Discussion

### Vitamin B_12_ biosynthesis potential of different bacteria

B vitamins play a key role in complex marine microbial interactions as they are obligatory cofactors in various essential metabolic reactions in all living organism [13, 14, 39–41]. An exciting fact about B_12_ is that genes for synthesis of this complex cofactor have never made the transition to the eukaryotic kingdom, although it is required by both prokaryotes and eukaryotes. *De novo* synthesis is restricted to a minor fraction of bacteria and archaea, thus, suggesting that the ability to synthesise B_12_ is disproportionate to its demand in nature [1, 4]. This phenomenon can be observed in various habitats, for example in the soil microbiome, where the proportion of B_12_ producers is less than one tenth [8]. Similar findings have been shown for the microbiome on human skin, where only 1% of the core species are predicted to produce B_12_ *de novo*, while 39 % of the species are predicted to use B_12_ for metabolism [42]. In order to adequately answer this fundamental question regarding the balance between B_12_ availability and consumption, we should aim to better understand the synthesis potential of individual prototrophic prokaryotes.

Here we present intra- and extracellular B_12_ concentrations of various B_12_ prototrophic, alphaproteobacterial strains. The concentration of intracellular B_12_ differs widely between the various heterotrophic bacteria examined. Converted, B_12_ molecules detected per cell ranged between 664 to 26,619 in the analysed bacterial cultures, including B_12_-provider and B_12_-retainer. Such strong variation in intracellular B_12_ concentrations have already been shown for a number of other prokaryotes, including Archaea, heterotrophic bacteria, and cyanobacteria [11, 34]. Also, in these studies, the detected intracellular B_12_ values differed up to three orders of magnitude and showed values similar to the ones we detected. Whether factors such as cell size, which we did not consider in our analysis, or the exact growth phase in which we took the samples had an influence on the strong variation cannot be clarified here. It is quite conceivable that different B_12_ requirements of the individual cells or different regulatory mechanisms of B_12_ synthesis played a decisive role for the intracellular B_12_ concentrations. Nevertheless, we can conclude that not only the genetic B_12_ biosynthetic potential within a microbial community is decisive, but rather which prokaryote is actually present is crucial for the availability of B_12_.

The extracellular concentrations of B_12_ detected in *M. algicola* and *P. inhibens* were about 8 and 256 times lower than respective intracellular levels. For example, *M. algicola* secreted about 936 B_12_ molecules per cell, which was roughly 85 times more as detected for *P. inhibens*. On the basis of the detected B_12_ demand of *T. pseudonana* determined by the bioassay, we can calculate that the eukaryote requires roughly 135,000 B_12_ molecules per cell, if we base the limitation of cell number solely on B_12_ availability. Thus, it would take about 144 living *M. algicola* cells that release B_12_ to cover the requirements for the growth of one *T. pseudonana* cell. In fact, the bacterial cell numbers in the stationary phase of the B_12_-provider-diatom co-cultures were at least 110 times higher than the cell numbers of *T. pseudonana*. These calculations are all based on ideal laboratory conditions, with sufficient supply of inorganic nutrients and organic substrates and may differ in natural environments where viral infections or sloppy feeding can lead to cell disruption and subsequent release of intracellular B_12_ [43, 44]. Also, B_12_ requirement of *T. pseudonana* cells can vary under different growth conditions. For example, it has been shown that growth of *T. pseudonana* even with 1 pM of B_12_ can result in a significant change in the metabolite pool of the diatom, which in turn may have implications for the interaction with bacteria [45]. Nevertheless, our data give a first approximate insight into the interplay between B_12_-producers and -consumers in the world of microorganisms.

### Bacterial effects on the growth of *T. pseudonana*

Growth characteristics of *T. pseudonana* in co-culture show not only the obligatory provision of B_12_ by bacteria but also other bacterial factors that influence growth. For example, we observed that *Sulfitobacter litoralis*, a representative of the Roseobacter group, showed inhibitory behaviour towards the diatom. Other studies have shown that Roseobacter group isolates can produce inhibitory substances, roseobacticides, which can suppress the growth of eukaryotic phototrophs [46]. The provision of B_12_ leads to a promotion in growth and, at the same time, growth of the diatom is inhibited. One reason for the different growth characteristics of the diatoms observed in co-culture with different bacteria could be the adaptation to different habitats where the bacterial isolates naturally occur.

In contrast to these observations, *Celeribacter baekdonensis* DSM 27375 significantly stimulated the growth of *T. pseudonana*. Even though *C. baekdonensis* did not provide B_12_ despite being synthesized, its presence in co-culture with B_12_ addition significantly increased the growth rate and growth yield of *T. pseudonana* compared to the positive control of the corresponding experimental run. In previous bacterial-diatom co-culture experiments, it has been shown that the excretion of cyclic peptides, diketopiperazines, by a bacterium, significantly increased diatom cell numbers [47]. Another plausible scenario is the synthesis and excretion of indoleacetic acid (IAA) by *C. baekdonensis*, which is a growth-promoting hormone for diatoms [48]. A similar effect is also conceivable for *C. baekdonensis* and would be exciting to explore in greater depth.

A finding that appears to be overlooked in the context of our actual question is the fact that the expected bacterial cell death does not necessarily lead to the release of B_12_, which would promote the growth of *T. pseudonana*, and thus promote the interaction. Even after up to six weeks in co-culture, we cannot observe significant growth of *T. pseudonana* despite the presence of a bacterial B_12_ prototroph. This fact highlights the importance of cell lysis mechanisms in nature, for example caused by viral infections or sloppy feeding. Already today, these two natural processes are considered to play a significant role in the turnover of dissolved organic matter [44, 49–51] and are likely to also have a decisive influence on the release of B-vitamins in marine ecosystems [23]. Additionally, *T. pseudonana* is known to secret a B_12_ binding protein under B_12_ deficient conditions that has an affinity constant of 2 × 10^11^ M^−1^. This protein might help them to acquire B_12_ from the surroundings, when it is released through bacterial cell lysis mechanism [52]. Other phytoplankton might also have a similar strategy to scavenge B_12_ from the environment. When intracellular B_12_ is considered as a reservoir for other B_12_ auxotrophic microorganisms, then, for example, already 19 *M. algicola* cells would be sufficient to enable the growth of one *T. pseudonana* cell.

### The vital cofactor B_12_ is not shared by all prototrophic bacteria

About half of the marine phytoplankton species are B_12_ auxotrophs and rely on prototrophic prokaryotes to obtain this essential vitamin [1, 53]. Several co-culture experiments have confirmed that individual marine bacterial isolates, mainly Alphaproteobacteria, enable phytoplankton species to overcome their auxotrophy by providing the essential cofactor [13–16, 27, 28]. In our study we hypothesised that not all B_12_ prototrophs share B_12_ with other microorganisms and to prove that we performed individual co-culture experiments between *T. pseudonana* and 33 B_12_ prototrophic bacteria. B_12_ prototrophy of the bacterial isolates was confirmed by their genetic ability to synthesize B_12_ (Supplementary table S2) and their ability to grow in B_12_-free medium. The results of our study support this hypothesis, as we were able to identify one group of bacteria that enables growth of *T. pseudonana* by the supply of the essential cofactor, B_12_-providers. On the other hand, we also identified a second group of B_12_ prototrophic bacteria that did not support the growth of the diatom, the B_12_-retainers. Moreover, while categorizing them into B_12_-providers and B_12_-retainers, we observed that there are species within one genus, such as *P. inhibens* and *P. galleciensis*, in which one is a B_12_-provider and the other is a B_12_-retainer, respectively, although both of them possess the necessary genes for B_12_ biosynthesis. Yet, the question remains why some bacteria share the cofactor, and others, despite an obligatory interaction enforced in co-culture, do not. In the following, we describe and discuss three scenarios that we consider plausible, whereby not only one scenario has to be correct, but rather all three can take place in the B_12_-retainer strains that we have identified.

First, biosynthesis of metabolites, such as the energetically costly B_12_ cofactor, are subject to intracellular regulation. Transcriptional regulation of the B_12_ biosynthesis pathway determines whether, and in what quantity B_12_ is synthesised in the cell. For example, sigma factors can alter the specificity of an RNA polymerase for a particular promoter, so that gene expression is enhanced or reduced [54]. In the case of the bacterial isolate *Propionibacterium* strain UF1, the riboswitch *cbiMCbl* was identified to regulate the gene expression of the *cobA* operon and thus controls B_12_ biosynthesis [55]. It is also known that sufficient availability of B_12_ can repress B_12_ biosynthesis gene expression in bacteria [56, 57]. In gram-negative proteobacteria as well as in cyanobacteria, for example, cobalamin (pseudocobalamin, in case of some bacteria) biosynthesis and B_12_ transport genes are regulated by inhibition of translation initiation, whereas in some gram-positive bacteria gene regulation proceeds by transcriptional antitermination [58]. The mechanisms described above are likely to also occur in the bacterial isolates that we tested. The large difference between the detected intracellular B_12_ concentrations could therefore be due to differences in gene regulation of the different bacteria and may also have had an influence on the release of B_12_ in the co-culture with *T. pseudonana*.

Second, cobalamin, which we referred to here as B_12_ for simplicity, belongs to a group of B_12_-like metabolites, called cobamides. Each cobamide differs in the lower ligand attached. For example, the common cobamide, cobalamin, which is bioavailable to most microorganisms, carries 5,6-dimethylbenzimidazol (DMB) as its lower ligand, whereas pseudocobalamin synthesised by cyanobacteria in high concentrations in the ocean and being less or not bioavailable to most microorganisms, has adenine attached as its lower ligand [11, 41, 59, 60]. In general, the lower ligands of cobamides can be divided into benzimidazoles, purines, and phenols, and more than a dozen cobamides and cobamide-analogs have already been discovered [61]. However, research into the synthesis and actual diversity of cobamides, especially in marine bacteria and archaea, is still in its infancy. In our study, we were unable to detect intracellular B_12_ in four out of eight bacterial B_12_-retainer strains, although the cell counts at the time of sampling should have been sufficient for its detection. However, as is generally the case, our LC-MS analysis only targets cobalamin (B_12_) with its different upper ligands (adenosyl-, cyano-, methyl-, and hydroxy-cobalamin). Therefore, we cannot exclude the possibility that the here studied bacteria synthesise different cobamides, which are possibly not or less bioavailable to *T. pseudonana*, and not covered by our analytical measurement method. This speculation was supported by the fact that one of these four B_12_- retainer strains, *Sulfitobacter* sp. DFL-23, does not possess the DMB synthesis gene *bluB* and there was no intracellular B_12_ detected in this strain (Supplementary table S2 and Table 2). Again, it is difficult to explain this phenomenon solely depending on the presence of annotated DMB synthesis gene, as for *Loktanella salsilacus* DSM 16199 no *bluB* gene was annotated, still we detected intracellular B_12_ in this strain using our detection method (Supplementary table S2 and Table 2).

Third, the bacteria we have identified as B_12_-retainer simply may not have actively released the synthesised B_12_ into their environment. Regardless of the importance of B_12_ for the vast majority of living organisms on our planet, its excretion mechanisms are to our knowledge still largely unknown. Its size of more than 1,350 Dalton does not allow sufficient diffusion through the cell membrane, which would enable microbial interactions [32]. Thus, it is likely that an unknown mechanism is required for its release. This assumption is further supported by the fact that we were able to detect intracellular B_12_ in four of the eight B_12_-retainer strains and at concentrations comparable to those detected in the B_12_-provider strains. In addition, we could detect intracellular B_12_ in *P. xiamenensis*, but none in its exometabolome. On the other hand, presence of extracellular B_12_ was detected in the exometabolome of both the provider strains examined, *M. algicola* and *P. inhibens*. Our findings show that not all bacteria share the pivotal cofactor with their environment, which has an impact on our current understanding of the marine B_12_ cycle and presumably in other ecosystems as well. The active exchange of B_12_ and thus microbial interaction plays a much smaller role than previously assumed for a relatively large number of bacteria. Consequently, for some of the B_12_ prototrophic bacteria within a community, it is likely that the cofactor is only released upon cell lysis.

### B_12_ production in the marine ecosystem and ecological implications

Looking at the original source of B_12_ in nature, namely prototrophic bacteria and archaea, the bacteria studied here show pronounced differences between the biosynthetic potentials of the cofactors and the ability to share them with their environment. Thus, the natural source of vitamin B_12_ within a given ecosystem does not primarily depend on the ratio of prototrophic bacteria, but even more crucially on how much of the cofactor is synthesised by the prototrophic prokaryotes within an ecosystem and is actively released. The fact that some bacteria do not voluntarily share B_12_ with ambient microorganisms, significantly increases the importance of processes, such as sloppy feeding by zooplankton or virus infections [44, 49–51], for the release of vitamins in the marine and likely also other ecosystems.

Our results also contribute to the controversially discussed question of whether B_12_ prototrophic bacteria live in symbiosis with phototrophic microorganisms [13, 30]. Despite numerous co-cultivation experiments demonstrating the obligatory provision of B_12_ by individual bacteria to phototrophic microorganisms, the decisive question of the mechanism of provision has so far been overlooked [13–16, 27, 28]. In our view, however, this question is crucial when assessing whether a symbiotic interaction is taking place. Our results support the hypothesis that a bacterial mechanism for the active release is likely to exist, as our experiments distinguish between B_12_-provider and B_12_-retainer within prototrophic bacteria. Looking at the ecological niches and the isolation sites of the two respective groups, differences can be identified. Most B_12_-provider strains were isolated from or discovered in association with eukaryotic microorganisms, whereas most B_12_-retainer strains were isolated as free-living in the ocean (Supplementary table S4). Moreover, six of the tested bacterial strains were isolated from dinoflagellates and five of them were B_12_-provider. Since we used a diatom as a B_12_ auxotrophic organism in our study, it would also be interesting to know if these B_12_-provider strains also provide B_12_ to other phytoplankton, such as dinoflagellates. Also, in this study we only studied bacteria from the alphaproteobacteria class, since a large share of them are known to be B_12_ prototrophs and abundant in the marine ecosystem. For future studies, it would be interesting to see if a similar pattern of B_12_ provisioning can be observed in bacteria from other classes. Our results indicate that the B_12_ prototrophy of a bacterium does not necessarily indicate a mutualistic interaction with other auxotrophic microorganisms. However, the bacterial group of B_12_-provider in particular seems to favour living in close proximity to other microorganisms, which is why the exchange of B_12_ for e.g. organic compounds can establish itself as a distinct symbiotic interaction between individual microorganisms.

## Supplementary information

Supplementry Table 1

Supplementry Table 2

Supplementry Table 3

Supplementry Table 4

Supplementry Figure 1

Supplementry Figure 2

Supplementry Figure 3

Supplementry Figure 4

Supplementry Figure 5

Supplementry Figure 6

Supplementry Figure 7

## Data Availability

The datasets generated during the current study are available from the corresponding author on reasonable request.
