# Supplementary material for: Vitamin B12 is not shared by all marine prototrophic bacteria with their environment
Source: ISME J. 2023 Mar 13;17(6):836–45. doi: 10.1038/s41396-023-01391-3 (PMC10203341; doi:10.1038/s41396-023-01391-3)
Supplement: Supplementary file 1 — Supplementry Table 1 [file 41396_2023_1391_MOESM1_ESM.docx]

Synchronized ASW- Medium (syn-ASW):

Dissolve the following salts in 900 ml milli-Q water

3.0 g Na_2_SO_4_ Sodium Sulfate

0.01 g KH_2_PO_4_ Monopotassium phosphate

0.025 g NH_4_Cl Ammonium chloride

22.0 g NaCl Sodium chloride

7.0 g MgCl_2_ x 6 H_2_O Magnesium chloride

0.7 g KCl Potassium Chloride

1.0 g CaCl_2_ x 2 H_2_O Calcium Chloride

0.098 g KBr Potassium Bromide

0.003 g H_3_BO_3_ Boric Acid

0.003 g NaF Sodium Fluoride (toxic)

0.024 g SrCl_2_ x 6 H_2_O Strontium Chloride

0.075 g NaNO_3_ Sodium nitrate

0.03 g Na_2_SiO_3_ x 9H_2_O Sodium silicate

(or 0.026 g Na_2_SiO_3_ x 5H_2_O)

Dissolve the following salt in 100 ml milli-Q water:

0.2 g NaHCO_3_

Autoclave both bottles apart from each other and mix them afterward.

Trace element solution (according to Zech et al. 2009)

3.15g FeCl_3_ x 6H_2_O Ferric chloride (FeIII)

5.2 g Na_2_EDTA (Titriplex) Disodium-ethyleneditetraacetic acid

0.18 g MnCl_2_ x 4H_2_O Manganese chloride tetrahydrate

0.19 g CoCl_2_ x 6H_2_O Cobalt chloride hexahydrate (toxic)

0.0126 g CuSO_4_ x 5H_2_O Copper sulfate pentahydrate

0.024 g NiCl_2_ x 6H_2_O Nickel chloride hexahydrate (toxic)

0.144 g ZnSO_4_ x 7H_2_O Zinc sulfate heptahydrate

0.036 g Na_2_MoO_4_ x 2H_2_O Sodium molybdate dihydrate

Add 1 ml trace element solution per 1l syn-ASW medium before autoclaving the medium.

Add 500 pM (picomole) concentration of each of vitamin B1, B2, B3, B5, B6, and B7 after autoclaving the medium.

**Table S1:** Listed is the recipe for the synchronized artificial seawater (syn-ASW)-medium used, which promotes the growth of *T. pseudonana* and the examined bacteria in equal measure.
