# Supplementary material for: Vitamin B12 is not shared by all marine prototrophic bacteria with their environment
Source: ISME J. 2023 Mar 13;17(6):836–45. doi: 10.1038/s41396-023-01391-3 (PMC10203341; doi:10.1038/s41396-023-01391-3)
Supplement: Supplementary file 3 — Supplementry Table 3 [file 41396_2023_1391_MOESM3_ESM.docx]

| Vitamin | Chemical Formula | Parent Ion *m/z* → Product Ions *m/z* (Collision Energies eV) | | Tube Lens (V) | |
| --- | --- | --- | --- | --- | --- |
| Cyanocobalamin (CB_12_) | C_63_H_88_CoN_14_O_14_P | 678.4 → | 358.1 (43), 997.5 (39), 147.0 (57) | | 135 |
| Adenosylcobalamin (AB_12_) | C_72_H_100_CoN_18_O_17_P | 790.5 → | 665.0 (39), 147.0 (59), 358.9 (54) | | 153 |
| Methylcobalamin (MB_12_) | C_63_H_91_CoN_13_O_14_P | 672.9 → | 665.3 (33), 146.9 (57), 971.5 (54) | | 138 |
| Hydroxycobalamin (HB_12_) | C_62_H_89_CoN_13_O_15_P | 673.8 → | 664.9 (10), 635.7 (33), 147.0 (53) | | 170 |

**Table S3:** Parameters for selected reaction monitoring mode on a TSQ Quantum Ultra triple quadrupole mass spectrometer (ThermoFisher Scientific).
