# Supplementary material for: Vitamin B12 is not shared by all marine prototrophic bacteria with their environment
Source: ISME J. 2023 Mar 13;17(6):836–45. doi: 10.1038/s41396-023-01391-3 (PMC10203341; doi:10.1038/s41396-023-01391-3)
Supplement: Supplementary file 4 — Supplementry Table 4 [file 41396_2023_1391_MOESM4_ESM.docx]

| Name of the strains | Strain ID | B_12_ Provider / Retainer | Motility | Isolation source | References (see list below) |
| --- | --- | --- | --- | --- | --- |
| *Antarctobacter heliothermus* EL-219 | DSM 11445 | Provider | No | Water sediment | Bartling et al. 2018 |
| *Dinoroseobacter shibae* | DSM 16493 | Provider | No | Dinoflagellate cultures of the Biological Research Institute of Helgoland | Bartling et al. 2018 |
| *Marinovum algicola* FF3 | DSM 10251 | Provider | Yes | Surface of marine Dinoflagellate *Prorocentrum lima* | Bartling et al. 2018 |
| *Nautella italica* R11 | DSM 26436 | Provider | Yes | Marine biofilm on stainless steel electrode | Bartling et al. 2018 |
| *Phaeobacter inhibens* | DSM 17395 | Provider | Yes | Seawater from larval cultures of the scallop *Pecten maximus* | Bartling et al. 2018 |
| *Phaeobacter inhibens* T5 | DSM 16374 | Provider | Yes | Water sample above an intertidal mud flat | Bartling et al. 2018 |
| *Ponticoccus litoralis* CL-GR66 | DSM 18986 | Provider | No | Coastal seawater | Hwang and Cho 2008 |
| *Aliiroseovarius crassostreae* CV919-312 | DSM 16950 | Provider | Yes | Eastern oyster *Crassostrea virginica* | Bartling et al. 2018 |
| *Roseovarius mucosus* DFL-24 | DSM 17069 | Provider | No | Culture of dinoflagellate *Alexandrium ostenfeldii* KO287 | Bartling et al. 2018 |
| *Ruegeria conchae* TW15 | DSM 29317 | Provider | Yes | Ark clam of *Scapharca broughtonii* | Bartling et al. 2018 |
| *Silicibacter* sp. | TM1040 | Provider | Yes | Culture of the dinoflagellate *Pfiesteria piscicida* CCMP1830 | Bartling et al. 2018 |
| *Sulfitobacter* sp. | DFL-14 | Provider | x | Dinoflagellate *Alexandrium ostenfeldii* | <https://img.jgi.doe.gov/> |
| *Thalassobius maritimus* GSW-M6 | DSM 28223 | Provider | Yes | Sea water | Bartling et al. 2018 |
| *Sulfitobacter* sp. | M22 | Provider | x | Sea water | Wienhausen et al. unpublished |
| *Roseovarius marinus* HDW-9 | DSM 25228 | Provider | No | Sea water, Yellow sea, Korea | Jung et al. 2011 |
| *Sulfitobacter mediterraneus* CH-B427 | DSM 12244 | Provider | Yes | Mediterranean Sea | Pukall et al. 1999 |
| *Roseovarius nubinhibens* ISM | DSM 15170 | Provider | Yes | Sea water, Caribbean Sea | Gonzalez et al. 2003 |
| *Thalassococcus halodurans* UST050418-052 | DSM 26915 | Provider | No | Surface of the marine sponge *Halichondria panicia* | Lee et al. 2007 |
| *Celeribacter baekdonensis* L-6 | DSM 27375 | Retainer | Yes | Sea water | Bartling et al. 2018 |
| *Pseudodongicola xiamenensis* Y-2 | DSM 18339 | Retainer | Yes | Oil-contaminated surface water | Bartling et al. 2018 |
| *Jannaschia helgolandensis* Hel10 | DSM 14858 | Retainer | No | Sea water | Bartling et al. 2018 |
| *Loktanella salsilacus* R-8904 | DSM 16199 | Retainer | No | Microbial mat | Bartling et al. 2018 |
| *Phaeobacter gallaeciensis* BS 107 | DSM 26640 | Retainer | Yes | Seawater from larval cultures of scallop *Pecten maximus* | Bartling et al. 2018 |
| *Sulfitobacter* sp. | DFL-23 | Retainer | x | Dinoflagellate *Alexandrium lusitanicum* | <https://img.jgi.doe.gov/> |

| Table S4 (continued) | | | | | |
| --- | --- | --- | --- | --- | --- |
| Name of the strains | Strain ID | B_12_ Provider / Retainer | Motility | Isolation source | References |
| *Sulfitobacter* sp. | M39 | Retainer | x | Sea water | Wienhausen et al. unpublished |
| *Loktanella* sp. | M215 | Retainer | x | Sea water | Wienhausen et al. unpublished |
| *Sulfitobacter* sp. | M220 | Retainer | x | Sea water | Wienhausen et al. unpublished |
| *Loktanella fryxellensis* | DSM 16213 | DSP | x | Microbial mat | <https://img.jgi.doe.gov/> |
| *Roseovarius halocynthiae* MA1-10 | DSM 27840 | DSP | Yes | Sea squirt *Halocynthia roretzi* | Kim et al. 2012 |
| *Roseovarius indicus* B108 | DSM 26383 | DSP | No | Deep sea water | Lai et al. 2011 |
| *Sulfitobacter pseudonitzschiae* H3 | DSM 26824 | DSP | No | Toxic marine diatom *Psudonitzschia multiseries* | Hong et al. 2015 |
| *Celeribacter neptunius* H14 | DSM 26471 | DSP | Yes | Sea water | Ivanova et al. 2010 |
| *Sulfitobacter litoralis* Iso 3 | DSM 17584 | Inhibitor | Yes | Sea water | Park et al. 2007 |
|  |  |  |  |  |  |

*x = Information not available, DSP= Different substrate preference

**References:**

1. Bartling P, Vollmers J, Petersen J. The first world swimming championships of roseobacters—phylogenomic insights into an exceptional motility phenotype. Syst Appl Microbiol. 2018; 41(6):544-54.
2. Hwang CY, Cho BC. *Ponticoccus litoralis* gen. nov., sp. nov., a marine bacterium in the family Rhodobacteraceae. Int J Syst Evol M. 2008; 58(6):1332-38.
3. <https://img.jgi.doe.gov/>
4. Wienhausen et al. unpublished.
5. Jung YT, Lee JS, Oh KH, Oh TK, Yoon JH. *Roseovarius marinus* sp. nov., isolated from seawater. Int J Syst Evol M. 2011; 61(2):427-32.
6. Pukall R, Buntefuß D, Frühling A, Rohde M, Kroppenstedt RM, Burghardt J, Lebaron P, Bernard L, Stackebrandt E. Sulfitobacter mediterraneus sp. nov., a new sulfite-oxidizing member of the α-Proteobacteria. Int J Syst Evol M. 1999; 49(2):513-19.
7. Gonzalez JM, Covert JS, Whitman WB, Henriksen JR, Mayer F, Scharf B, Schmitt R, Buchan A, Fuhrman JA, Kiene RP, Moran MA. *Silicibacter pomeroyi* sp. nov. and *Roseovarius nubinhibens* sp. nov., dimethylsulfoniopropionate-demethylating bacteria from marine environments. Int J Syst Evol M. 2003; 53(5):1261-69.
8. Lee OO, Tsoi MM, Li X, Wong PK, Qian PY. *Thalassococcus halodurans* gen. nov., sp. nov., a novel halotolerant member of the Roseobacter clade isolated from the marine sponge *Halichondria panicea* at Friday Harbor, USA. Int J Syst Evol M. 2007; 57(8):1919-24.
9. Kim YO, Kong HJ, Park S, Kang SJ, Kim WJ, Kim KK, Oh TK, Yoon JH. *Roseovarius halocynthiae* sp. nov., isolated from the sea squirt *Halocynthia roretzi*. Int J Syst Evol M. 2012; 62:931-36.
10. Lai Q, Zhong H, Wang J, Yuan J, Sun F, Wang L, Zheng T, Shao Z. *Roseovarius indicus* sp. nov., isolated from deep-sea water of the Indian Ocean. Int J Syst Evol M. 2011; 61(9):2040-44.
11. Hong Z, Lai Q, Luo Q, Jiang S, Zhu R, Liang J, Gao Y. *Sulfitobacter pseudonitzschiae* sp. nov., isolated from the toxic marine diatom *Pseudo-nitzschia multiseries*. Int J Syst Evol M. 2015; 65:95-100.
12. Ivanova EP, Webb H, Christen R, Zhukova NV, Kurilenko VV, Kalinovskaya NI, Crawford RJ. *Celeribacter neptunius* gen. nov., sp. nov., a new member of the class Alphaproteobacteria. Int J Syst Evol M. 2010; 60(7):1620-25.
13. Park JR, Bae JW, Nam YD, Chang HW, Kwon HY, Quan ZX, Park YH. *Sulfitobacter litoralis* sp. nov., a marine bacterium isolated from the East Sea, Korea. Int J Syst Evol M. 2007; 57(4):692-95.
14. Lau SC, Tsoi MM, Li X, Plakhotnikova I, Wu M, Wong PK, Qian PY. *Loktanella hongkongensis* sp. nov., a novel member of the α-Proteobacteria originating from marine biofilms in Hong Kong waters. Int J Syst Evol M. 2004; 54(6):2281-84.

**Table S4:** Information about the biogeography and motility of the B_12_-provider and B_12_-retainer strains used in this study.
