# Supplementary material for: Vitamin B12 is not shared by all marine prototrophic bacteria with their environment
Source: ISME J. 2023 Mar 13;17(6):836–45. doi: 10.1038/s41396-023-01391-3 (PMC10203341; doi:10.1038/s41396-023-01391-3)
Supplement: Supplementary file 5 — Supplementry Figure 1 [file 41396_2023_1391_MOESM5_ESM.pdf]

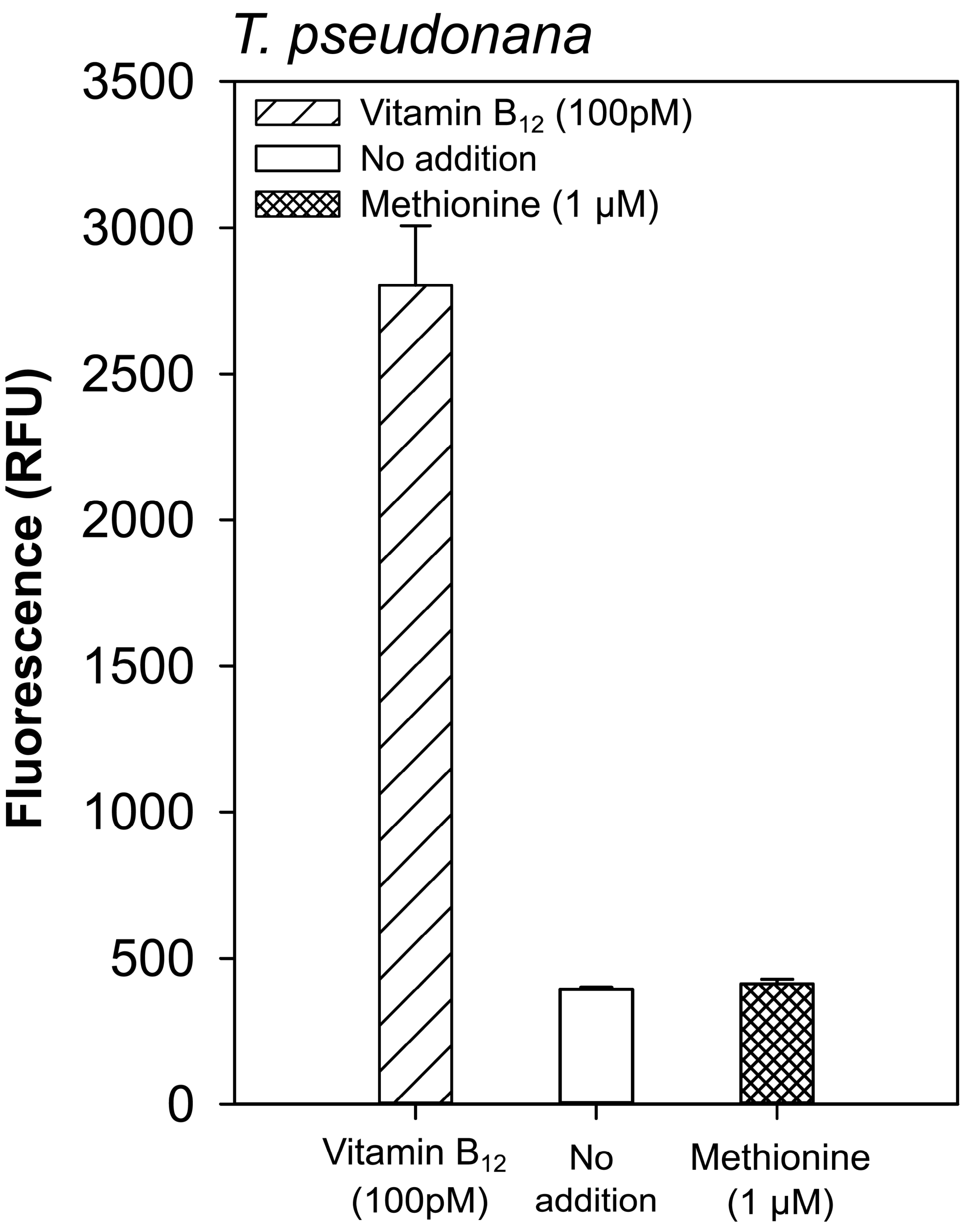

Supplementry Figure 1: Presented here is the maximum growth (determined by the relative fluorescence unit) in monocultures of *T. pseudonana* with the addition of B_{12} (100 pM), methionine (1 μM) and without any additive.
