# Supplementary material for: Vitamin B12 is not shared by all marine prototrophic bacteria with their environment
Source: ISME J. 2023 Mar 13;17(6):836–45. doi: 10.1038/s41396-023-01391-3 (PMC10203341; doi:10.1038/s41396-023-01391-3)
Supplement: Supplementary file 6 — Supplementry Figure 2 [file 41396_2023_1391_MOESM6_ESM.pdf]

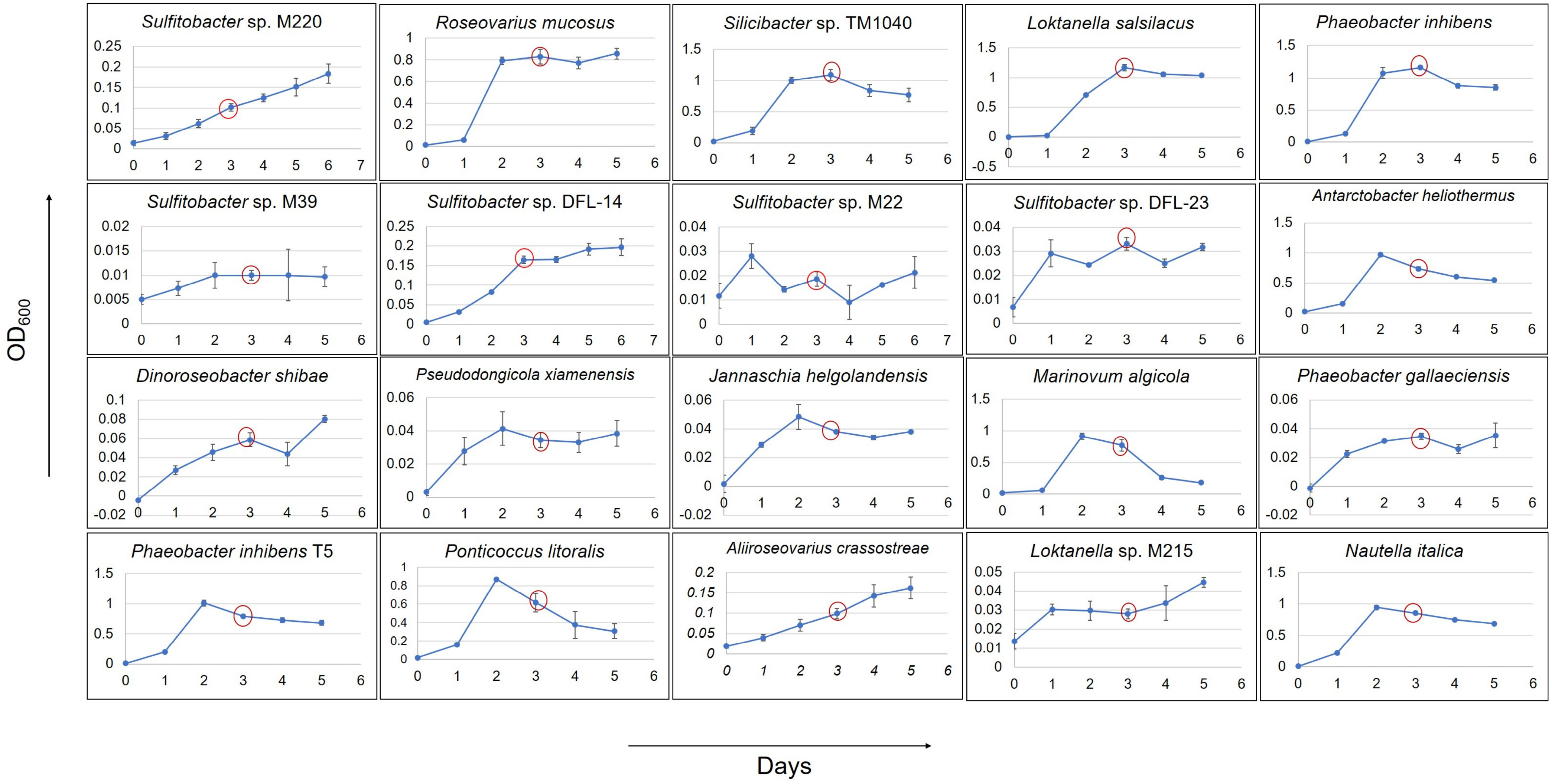

Supplementry Figure 2: Shown are the mean growth curves of all bacterial strains in mono-culture from which the intracellular and extracellular B_{12} concentrations were measured. Growth was monitored over time (days) using optical density (OD_{600}). The red circle represents the time of sampling for intracellular and extracellular B_{12} detection.
