# Supplementary material for: Vitamin B12 is not shared by all marine prototrophic bacteria with their environment
Source: ISME J. 2023 Mar 13;17(6):836–45. doi: 10.1038/s41396-023-01391-3 (PMC10203341; doi:10.1038/s41396-023-01391-3)
Supplement: Supplementary file 7 — Supplementry Figure 3 [file 41396_2023_1391_MOESM7_ESM.pdf]

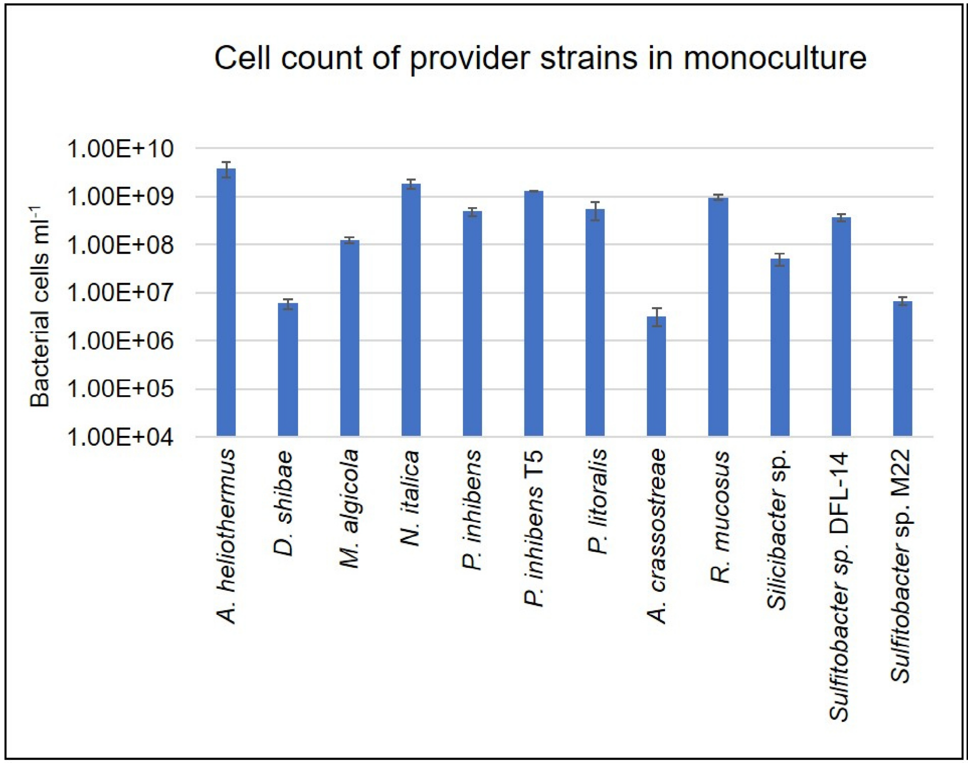

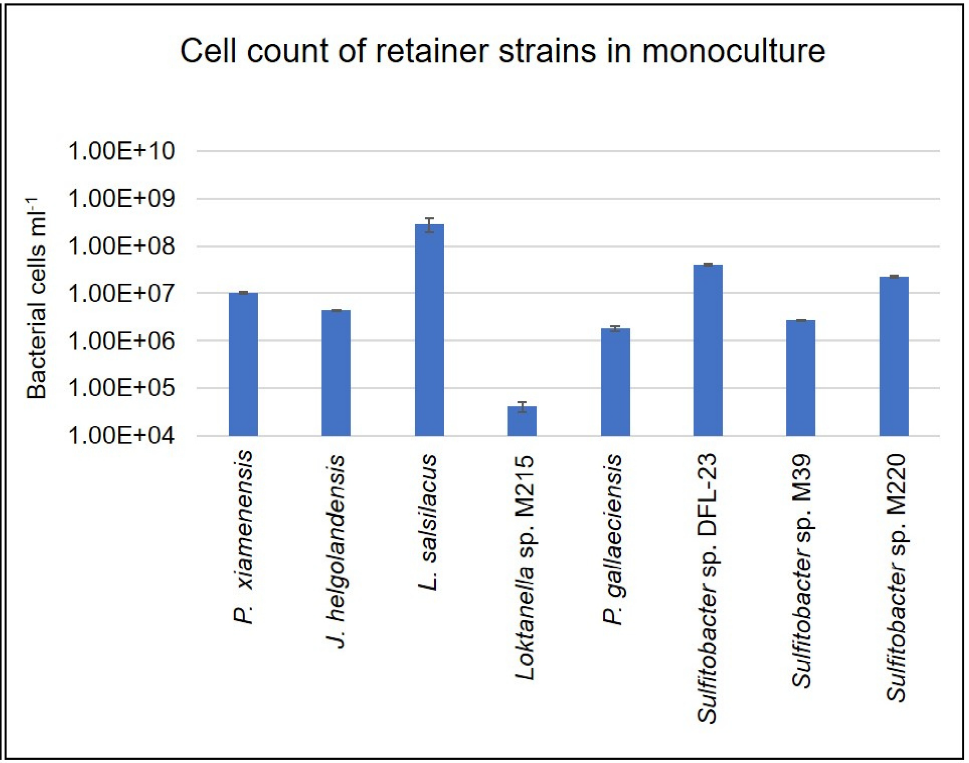

Supplementry Figure 3: Shown here are the cell counts measured by flow cytometry at the time of sampling for the detection of intracellular and extracellular B_{12} concentrations of prototrophic B_{12} bacteria in monocultures.
