# Supplementary material for: Vitamin B12 is not shared by all marine prototrophic bacteria with their environment
Source: ISME J. 2023 Mar 13;17(6):836–45. doi: 10.1038/s41396-023-01391-3 (PMC10203341; doi:10.1038/s41396-023-01391-3)
Supplement: Supplementary file 8 — Supplementry Figure 4 [file 41396_2023_1391_MOESM8_ESM.pdf]

1

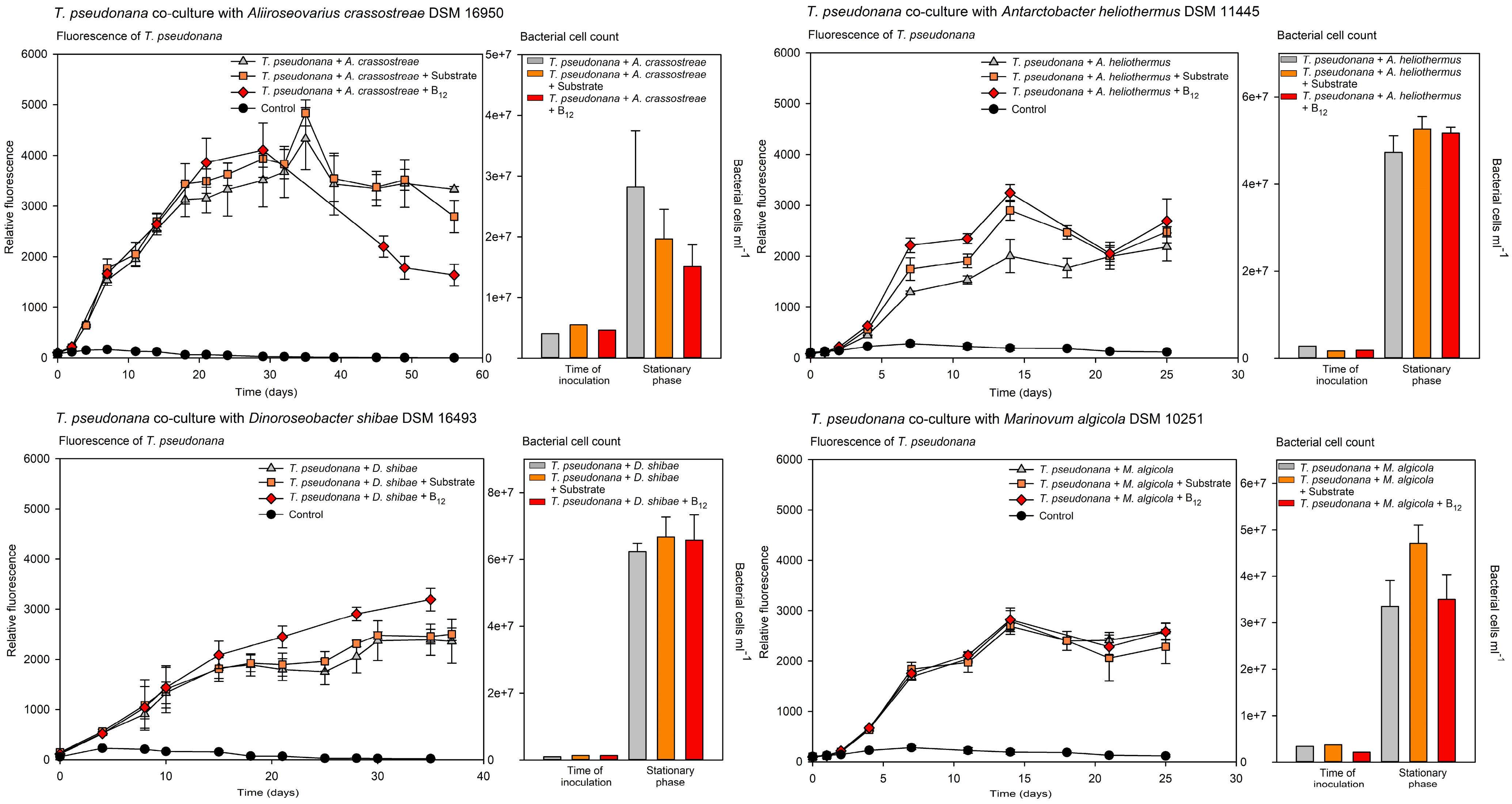

T. pseudonana co-culture with Aliiroseovarius crassostreae DSM 16950
Fluorescence of T. pseudonana
Relative fluorescence
Time (days)
T. pseudonana + A. crassostreae
T. pseudonana + A. crassostreae + Substrate
T. pseudonana + A. crassostreae + B12
Control
Bacterial cell count
Bacterial cells ml-1
Time of inoculation
Stationary phase
T. pseudonana co-culture with Antarctobacter heliothermus DSM 11445
Fluorescence of T. pseudonana
T. pseudonana + A. heliothermus
T. pseudonana + A. heliothermus + Substrate
T. pseudonana + A. heliothermus + B12
Control
Bacterial cell count
T. pseudonana co-culture with Dinoroseobacter shibae DSM 16493
Fluorescence of T. pseudonana
T. pseudonana + D. shibae
T. pseudonana + D. shibae + Substrate
T. pseudonana + D. shibae + B12
Control
Bacterial cell count
T. pseudonana co-culture with Marinovum algicola DSM 10251
Fluorescence of T. pseudonana
T. pseudonana + M. algicola
T. pseudonana + M. algicola + Substrate
T. pseudonana + M. algicola + B12
Control
Bacterial cell count

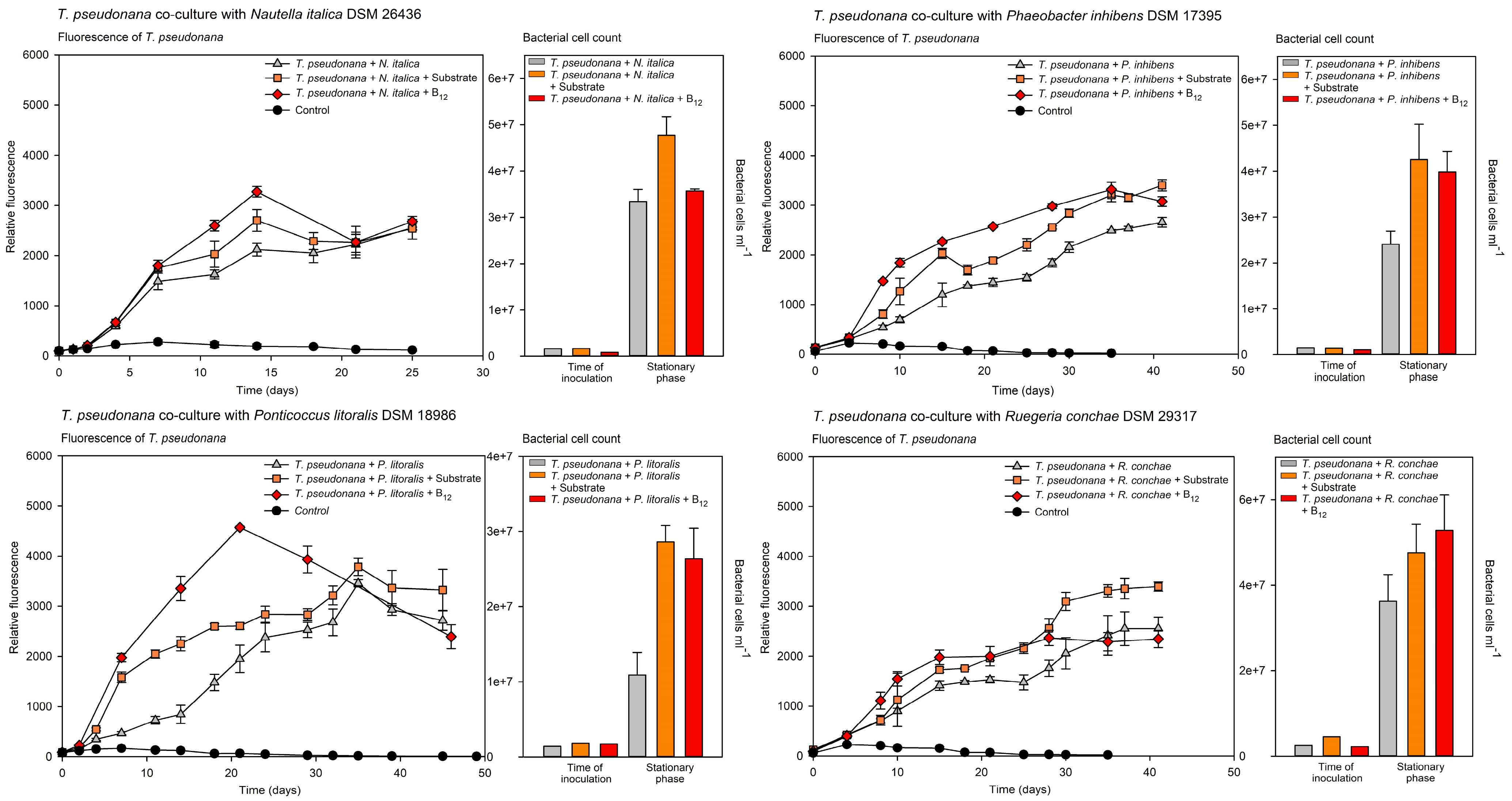

T. pseudonana co-culture with Nautella italica DSM 26436
Fluorescence of T. pseudonana
Relative fluorescence
Time (days)
T. pseudonana + N. italica
T. pseudonana + N. italica + Substrate
T. pseudonana + N. italica + B12
Control
Bacterial cell count
Bacterial cells ml-1
T. pseudonana + N. italica + Substrate
Time of inoculation
Stationary phase
T. pseudonana co-culture with Phaeobacter inhibens DSM 17395
T. pseudonana + P. inhibens
T. pseudonana + P. inhibens + Substrate
T. pseudonana + P. inhibens + B12
T. pseudonana co-culture with Ponticoccus litoralis DSM 18986
T. pseudonana + P. litoralis
T. pseudonana + P. litoralis + Substrate
T. pseudonana + P. litoralis + B12
T. pseudonana co-culture with Ruegeria conchae DSM 29317
T. pseudonana + R. conchae
T. pseudonana + R. conchae + Substrate
T. pseudonana + R. conchae + B12

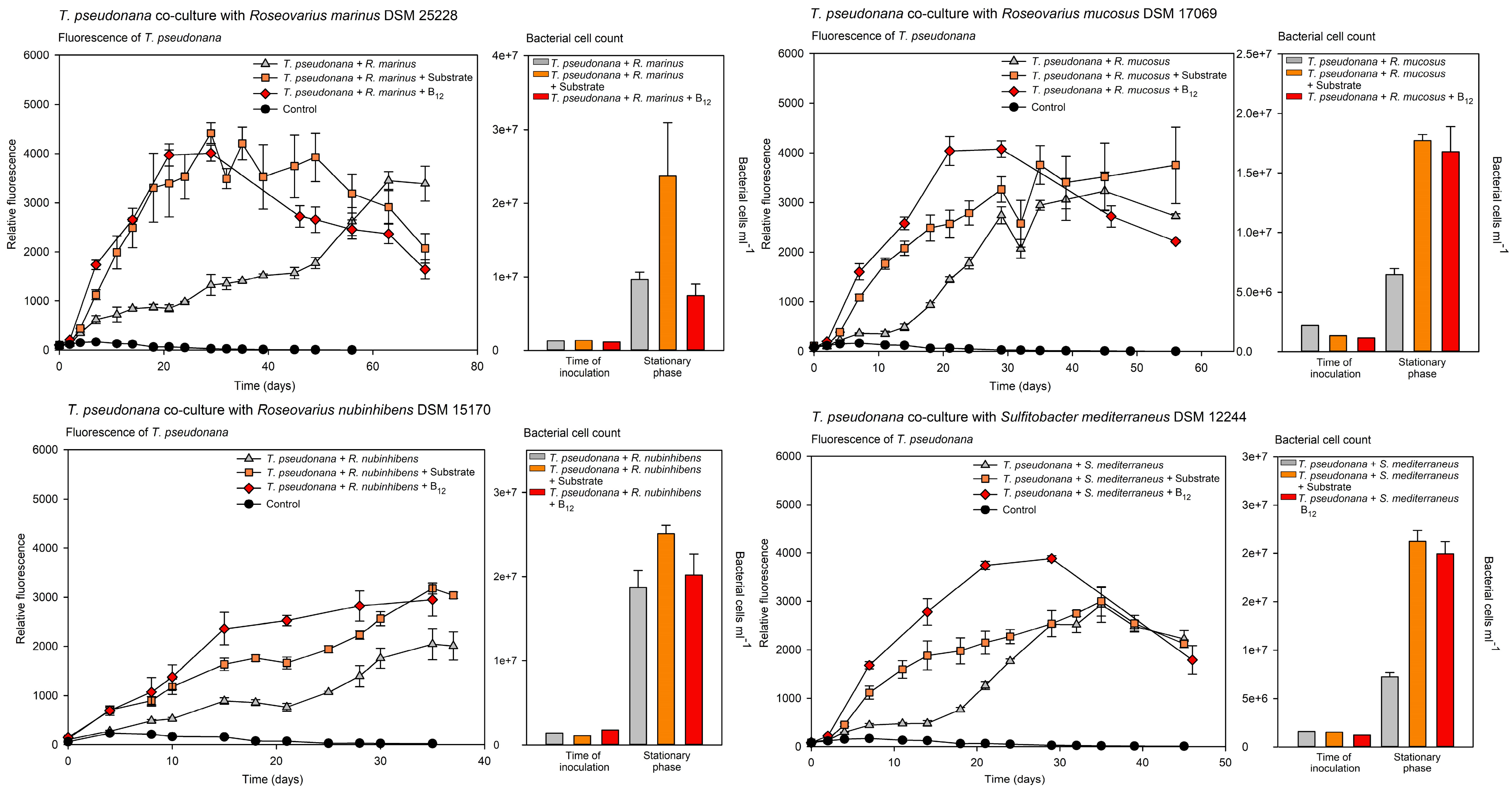
T. pseudonana co-culture with Roseovarius marinus DSM 25228
Fluorescence of T. pseudonana
Relative fluorescence
Time (days)
T. pseudonana + R. marinus
T. pseudonana + R. marinus + Substrate
T. pseudonana + R. marinus + B12
Control
Bacterial cell count
Bacterial cells ml-1
Time of inoculation
Stationary phase
T. pseudonana co-culture with Roseovarius mucosus DSM 17069
T. pseudonana + R. mucosus
T. pseudonana + R. mucosus + Substrate
T. pseudonana + R. mucosus + B12
T. pseudonana co-culture with Roseovarius nubinhibens DSM 15170
T. pseudonana + R. nubinhibens
T. pseudonana + R. nubinhibens + Substrate
T. pseudonana + R. nubinhibens + B12
T. pseudonana co-culture with Sulfitobacter mediterraneus DSM 12244
T. pseudonana + S. mediterraneus
T. pseudonana + S. mediterraneus + Substrate
T. pseudonana + S. mediterraneus + B12

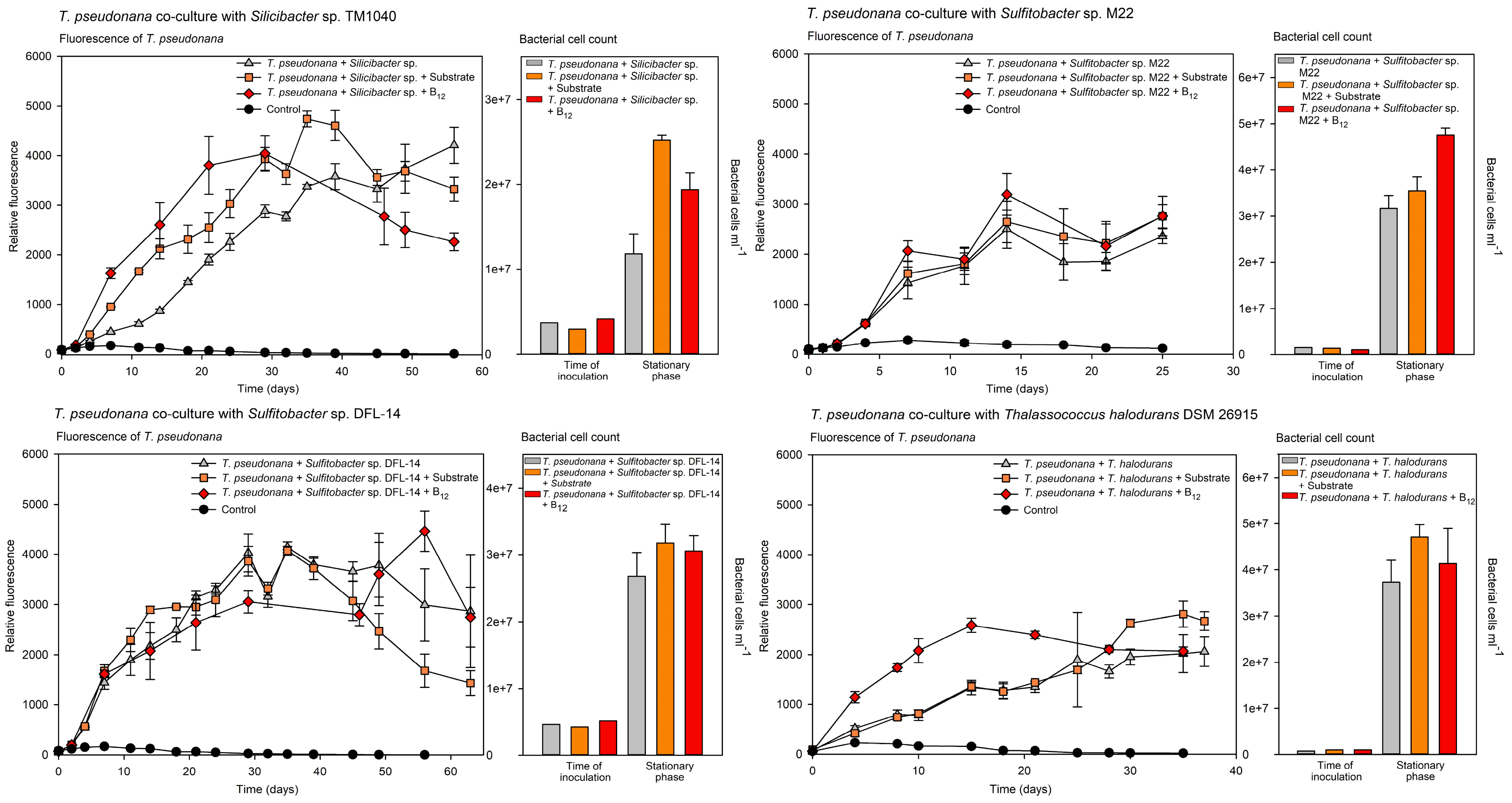
T. pseudonana co-culture with Silicibacter sp. TM1040
Fluorescence of T. pseudonana
Relative fluorescence
Time (days)
T. pseudonana + Silicibacter sp.
T. pseudonana + Silicibacter sp. + Substrate
T. pseudonana + Silicibacter sp. + B12
Control
Bacterial cell count
T. pseudonana + Silicibacter sp.
T. pseudonana + Silicibacter sp. + Substrate
T. pseudonana + Silicibacter sp. + B12
Bacterial cells ml-1
Time of inoculation
Stationary phase
T. pseudonana co-culture with Sulfitobacter sp. M22
Fluorescence of T. pseudonana
T. pseudonana + Sulfitobacter sp. M22
T. pseudonana + Sulfitobacter sp. M22 + Substrate
T. pseudonana + Sulfitobacter sp. M22 + B12
Control
Bacterial cell count
T. pseudonana + Sulfitobacter sp. M22
T. pseudonana + Sulfitobacter sp. M22 + Substrate
T. pseudonana + Sulfitobacter sp. M22 + B12
T. pseudonana co-culture with Sulfitobacter sp. DFL-14
Fluorescence of T. pseudonana
T. pseudonana + Sulfitobacter sp. DFL-14
T. pseudonana + Sulfitobacter sp. DFL-14 + Substrate
T. pseudonana + Sulfitobacter sp. DFL-14 + B12
Control
Bacterial cell count
T. pseudonana + Sulfitobacter sp. DFL-14
T. pseudonana + Sulfitobacter sp. DFL-14 + Substrate
T. pseudonana + Sulfitobacter sp. DFL-14 + B12
T. pseudonana co-culture with Thalassococcus halodurans DSM 26915
Fluorescence of T. pseudonana
T. pseudonana + T. halodurans
T. pseudonana + T. halodurans + Substrate
T. pseudonana + T. halodurans + B12
Control
Bacterial cell count
T. pseudonana + T. halodurans
T. pseudonana + T. halodurans + Substrate
T. pseudonana + T. halodurans + B12

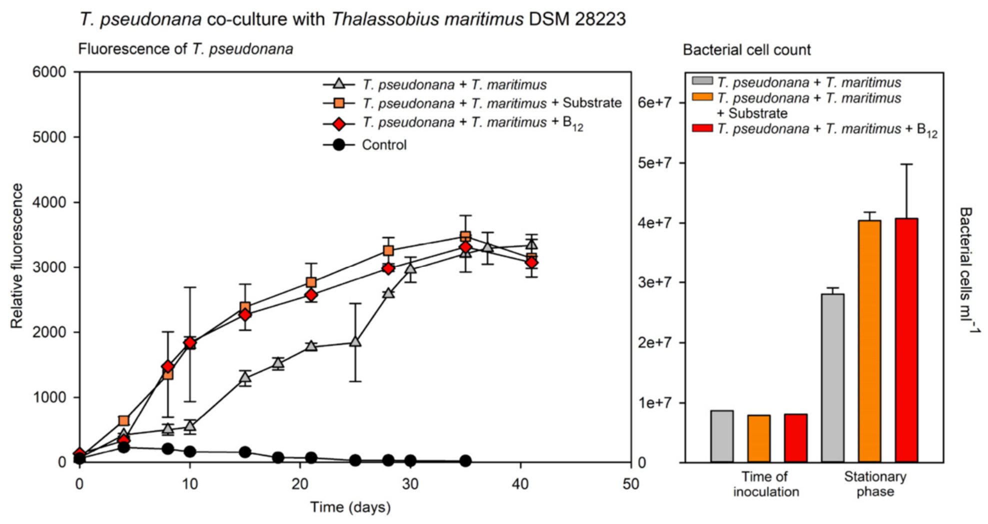

Supplementry Figure 4: Depicted are co-cultures of *T. pseudonana* with B_{12}-provider strains. (left panels; growth curves) Growth of *T. pseudonana* in co-culture monitored by relative fluorescence unit (RFU) over time with additions of substrate mix (orange square), B_{12} (red diamond) or without addition of either (grey triangle). (Right panels; bar plots) Bacterial cell counts in co-cultures at the time of inoculation and early stationary growth phase of *T. pseudonana*.
