# Supplementary material for: Vitamin B12 is not shared by all marine prototrophic bacteria with their environment
Source: ISME J. 2023 Mar 13;17(6):836–45. doi: 10.1038/s41396-023-01391-3 (PMC10203341; doi:10.1038/s41396-023-01391-3)
Supplement: Supplementary file 9 — Supplementry Figure 5 [file 41396_2023_1391_MOESM9_ESM.pdf]

1

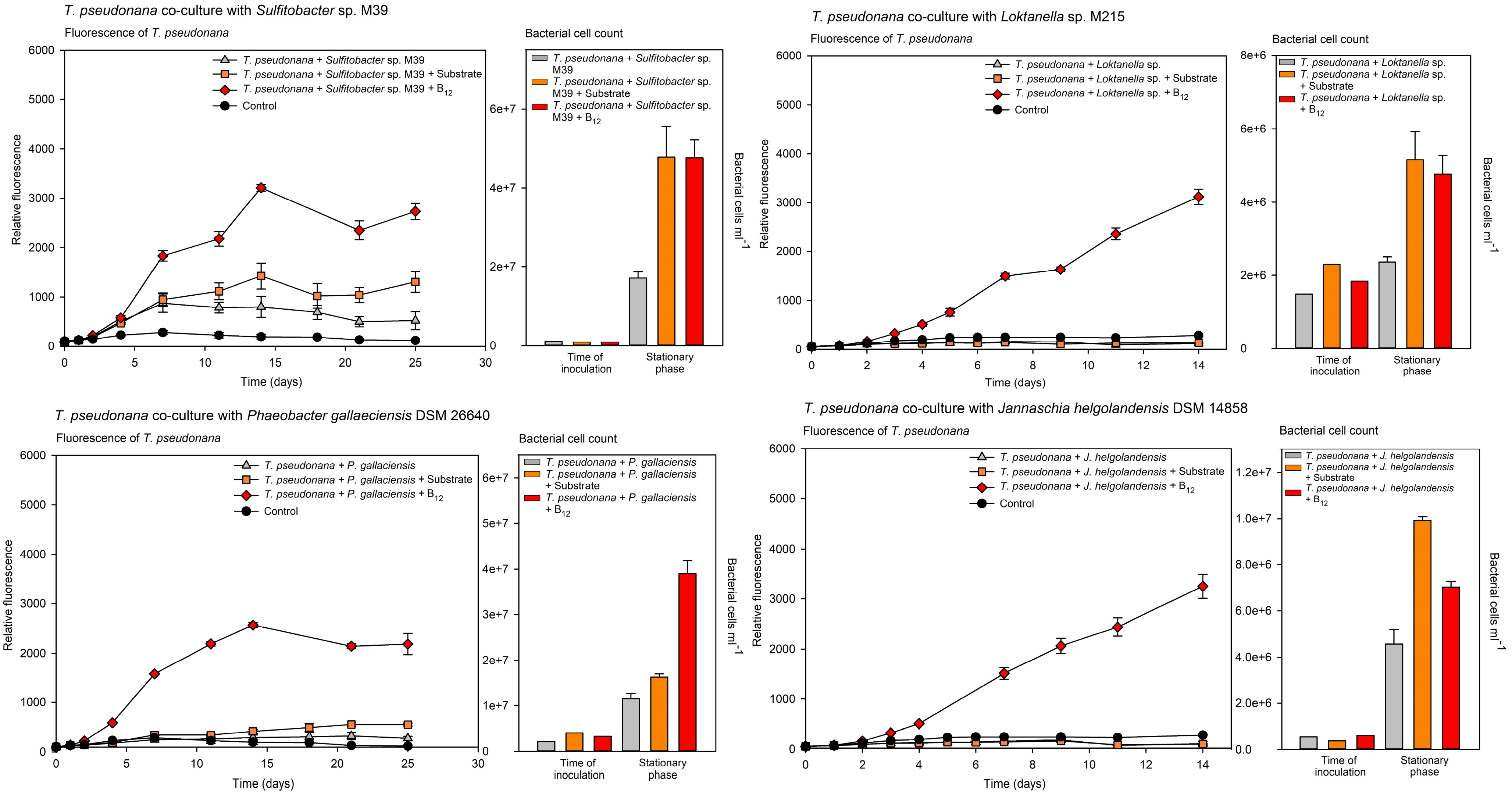

T. pseudonana co-culture with Sulfitobacter sp. M39
Fluorescence of T. pseudonana
T. pseudonana + Sulfitobacter sp. M39
T. pseudonana + Sulfitobacter sp. M39 + Substrate
T. pseudonana + Sulfitobacter sp. M39 + B12
Control
Relative fluorescence
Time (days)
Bacterial cell count
T. pseudonana + Sulfitobacter sp. M39
T. pseudonana + Sulfitobacter sp. M39 + Substrate
T. pseudonana + Sulfitobacter sp. M39 + B12
Bacterial cells ml-1
Time of inoculation
Stationary phase
T. pseudonana co-culture with Loktanella sp. M215
Fluorescence of T. pseudonana
T. pseudonana + Loktanella sp.
T. pseudonana + Loktanella sp. + Substrate
T. pseudonana + Loktanella sp. + B12
Control
Relative fluorescence
Time (days)
Bacterial cell count
T. pseudonana + Loktanella sp.
T. pseudonana + Loktanella sp. + Substrate
T. pseudonana + Loktanella sp. + B12
Bacterial cells ml-1
Time of inoculation
Stationary phase
T. pseudonana co-culture with Phaeobacter gallaeciensis DSM 26640
Fluorescence of T. pseudonana
T. pseudonana + P. gallaciensis
T. pseudonana + P. gallaciensis + Substrate
T. pseudonana + P. gallaciensis + B12
Control
Relative fluorescence
Time (days)
Bacterial cell count
T. pseudonana + P. gallaciensis
T. pseudonana + P. gallaciensis + Substrate
T. pseudonana + P. gallaciensis + B12
Bacterial cells ml-1
Time of inoculation
Stationary phase
T. pseudonana co-culture with Jannaschia helgolandensis DSM 14858
Fluorescence of T. pseudonana
T. pseudonana + J. helgolandensis
T. pseudonana + J. helgolandensis + Substrate
T. pseudonana + J. helgolandensis + B12
Control
Relative fluorescence
Time (days)
Bacterial cell count
T. pseudonana + J. helgolandensis
T. pseudonana + J. helgolandensis + Substrate
T. pseudonana + J. helgolandensis + B12
Bacterial cells ml-1
Time of inoculation
Stationary phase

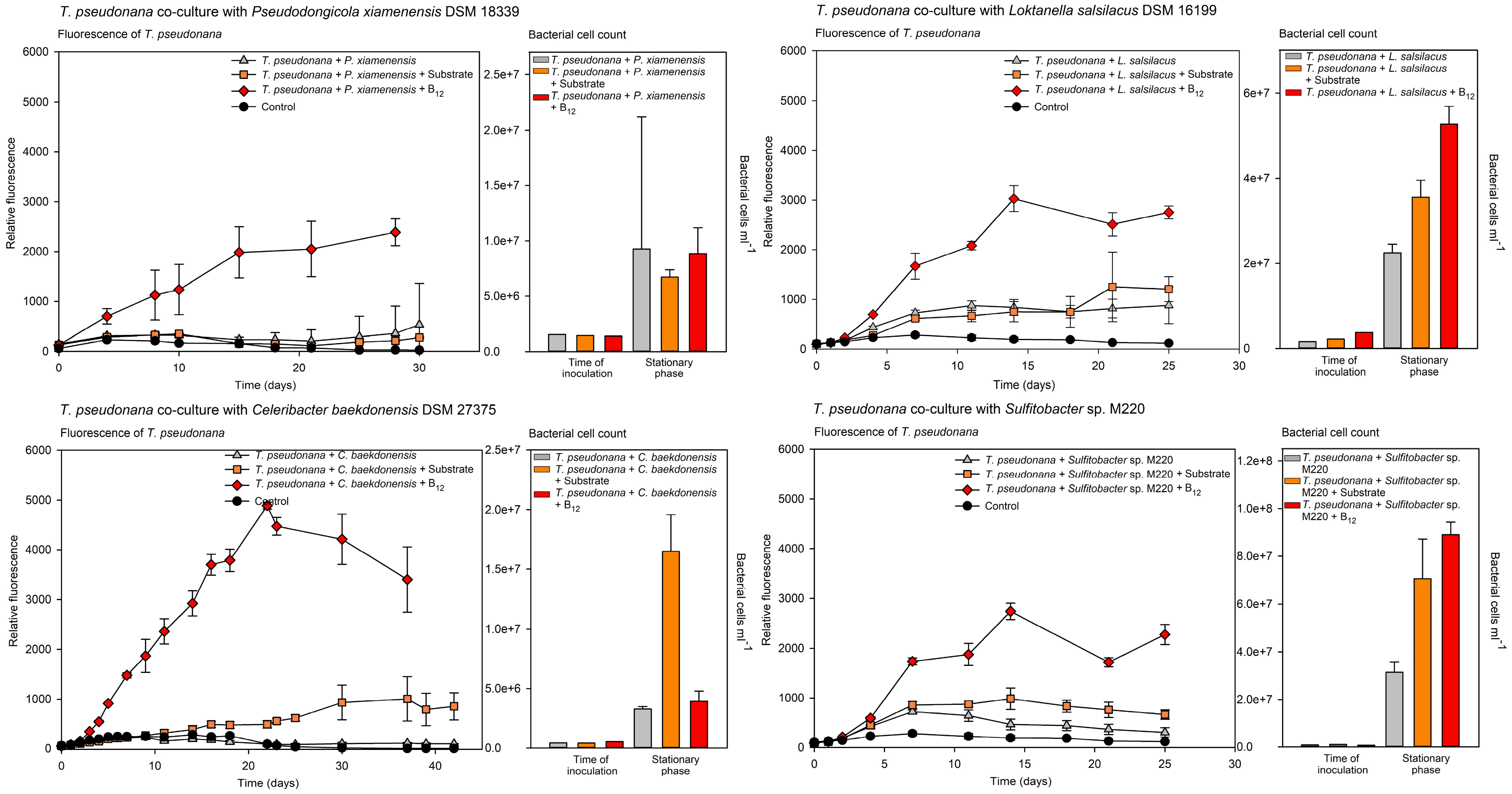

Supplementry Figure 5: Depicted are co-cultures of *T. pseudonana* with B_{12}-retainer strains. (left panels; growth curves) Growth of *T. pseudonana* in co-culture monitored by relative fluorescence unit (RFU) over time with additions of substrate mix (orange square), B_{12} (red diamond) or without addition of either (grey triangle). (Right panels; bar plots) Bacterial cell counts in co-cultures at the time of inoculation and early stationary growth phase of *T. pseudonana*.
