# Supplementary material for: Vitamin B12 is not shared by all marine prototrophic bacteria with their environment
Source: ISME J. 2023 Mar 13;17(6):836–45. doi: 10.1038/s41396-023-01391-3 (PMC10203341; doi:10.1038/s41396-023-01391-3)
Supplement: Supplementary file 10 — Supplementry Figure 6 [file 41396_2023_1391_MOESM10_ESM.pdf]

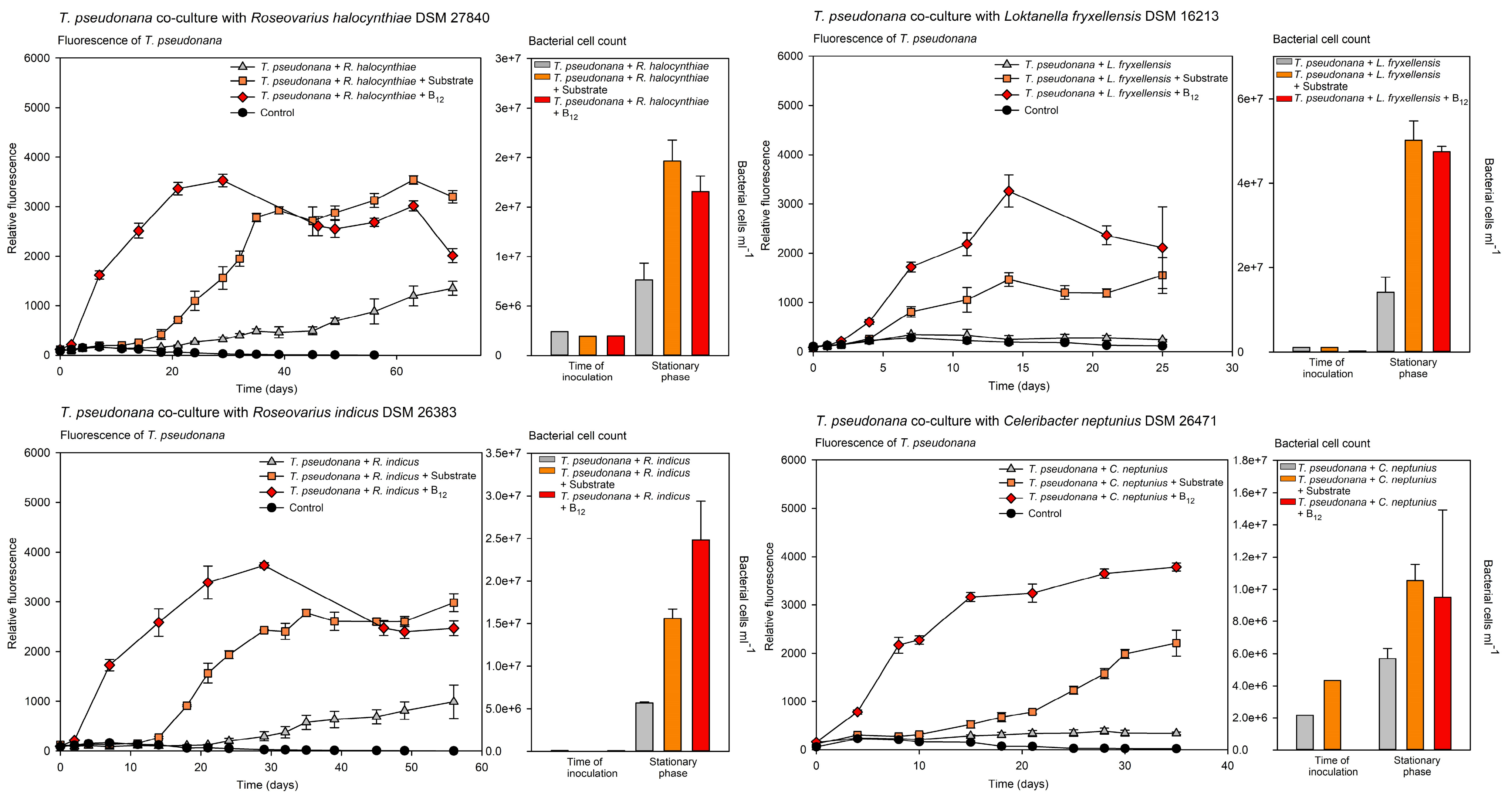

Supplementry Figure 6: Depicted are co-cultures of *T. pseudonana* with B_{12}-prototrophic strains that only promote the growth of *T. pseudonana* with substrate additions. (left panels; growth curves) Growth of *T. pseudonana* in co-culture monitored by relative fluorescence unit (RFU) over time with additions of substrate mix (orange square), B_{12} (red diamond) or without addition of either (grey triangle). (Right panels; bar plots) Bacterial cell counts in co-cultures at the time of inoculation and early stationary growth phase of *T. pseudonana*.
