# Supplementary material for: Vitamin B12 is not shared by all marine prototrophic bacteria with their environment
Source: ISME J. 2023 Mar 13;17(6):836–45. doi: 10.1038/s41396-023-01391-3 (PMC10203341; doi:10.1038/s41396-023-01391-3)
Supplement: Supplementary file 11 — Supplementry Figure 7 [file 41396_2023_1391_MOESM11_ESM.pdf]

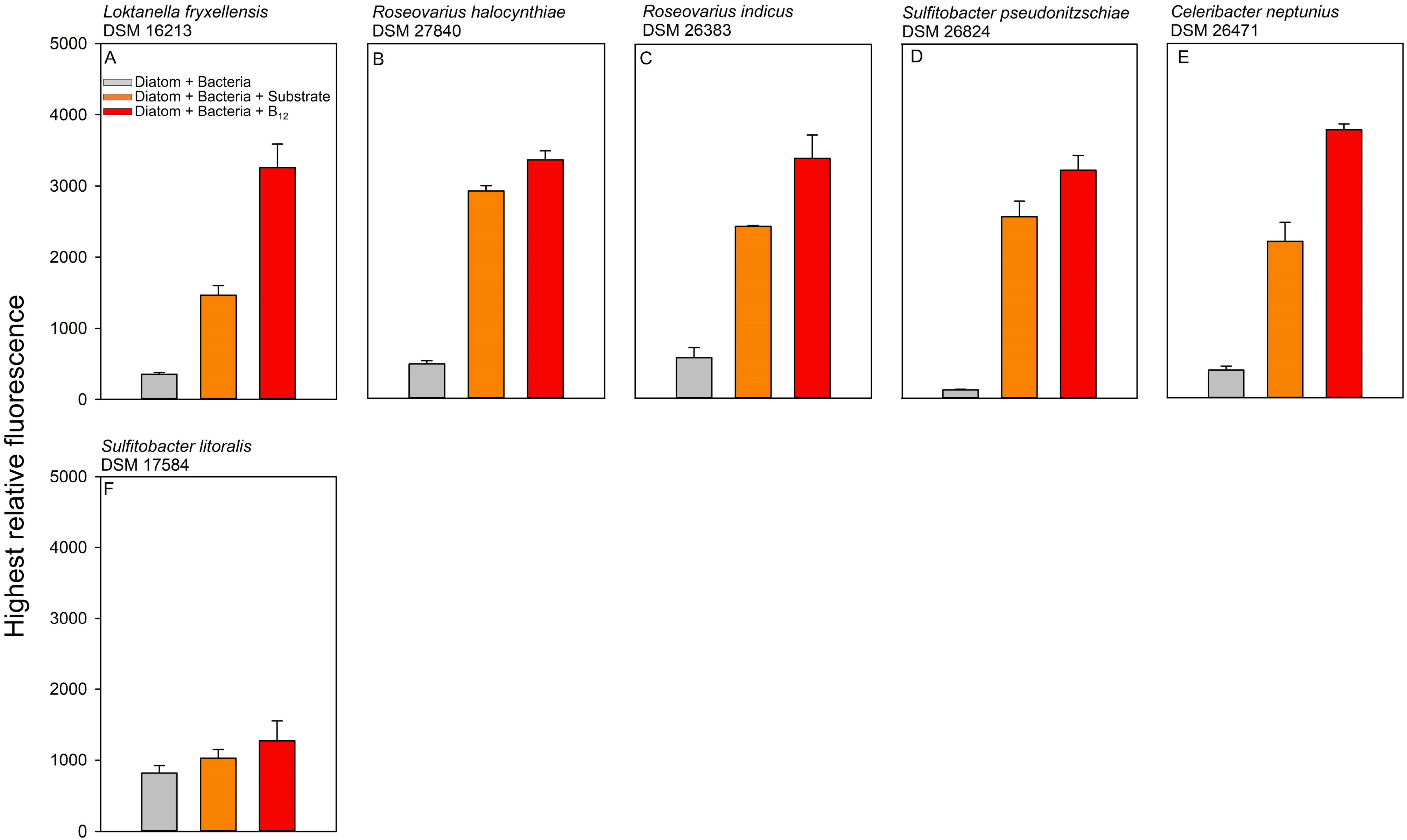

Supplementry Figure 7: Bars represent the maximum relative fluorescence of *T. pseudonana* during growth in co-culture with 5 different bacteria that provides B_{12} only with the addition of substrate (A-E) (corresponding growth curves can be seen in Figure S6) and with *S. litoralis* which inhibited the growth of the diatom (F) under different growth conditions. Grey bars represent maximum relative fluorescence of *T. pseudonana* in co-cultures without further additions, orange co-cultures with an additional substrate mix and red the co-cultures with B_{12} additions.
